# Supplementary material for: PCNA Retention on DNA into G2/M Phase Causes Genome Instability in Cells Lacking Elg1
Source: Cell Rep. 2016 Jun 30;16(3):684–95. doi: 10.1016/j.celrep.2016.06.030 (PMC4956615; doi:10.1016/j.celrep.2016.06.030)
Supplement: Document S1. Supplemental Experimental Procedures, Figures S1–S4, and Table S1 [file mmc1.pdf]

Cell Reports, Volume *16*

Supplemental Information

PCNA Retention on DNA into G2/M Phase Causes Genome Instability in Cells Lacking Elg1

Catherine Johnson, Vamsi K. Gali, Tatsuro S. Takahashi, and Takashi Kubota

Supplemental Experimental Procedures

Plasmid construction

The plasmids used are listed in Table S1. The plasmid pTK23 containing *NHP6B*, *TRP1* and *POL30* was constructed by fusing PCR products amplified from genomic DNA and the backbone of pRS304 using the In-fusion Cloning kit (Takara Clontech). Similarly, the plasmid pTK24 containing *NHP6B*, *LEU2*, and *POL30* was constructed using the In-fusion Cloning kit. For constructing the plasmids containing PCNA mutant alleles (pTK26, 27, 28, and 30), *pol30* mutant alleles were amplified from genomic DNA prepared from existing *pol30* mutants (Goellner et al., 2014; Windecker and Ulrich, 2008). To construct the plasmid pTK31 for M/G1-tagging, the PCR fragment containing the Sic1 promoter (688 bp upstream of ATG) and the coding region of the N-terminus of Sic1 (1-105 amino acids) was fused to the plasmid backbone of pRDK1597 (Hombauer et al., 2011) (including natNT2, excluding S-tag) using the In-fusion Cloning kit. The plasmid pRS303-*GALp-POL30-ADH1t* was constructed by fusing PCR fragments of *POL30* and the terminator of *ADH1* to pRS303-*GALp* using the In-fusion Cloning kit.

Yeast strains

S. cerevisiae strains used are listed in Table S2. Epitope tagging and gene disruption were carried out using standard PCR-based gene-insertion methods (Longtine et al., 1998). Yeast strains expressing trimer interface PCNA mutants (Goellner et al., 2014) were gifted from the Kolodner lab, and were used for initial screening for the MMS sensitivity and their retentions on chromatin (Figure 3B and Figure S2C). Since those strains have a disrupted *NHP6B* gene (located upstream of the *POL30* locus) caused

by insertion of the *LEU2* gene, we constructed strains expressing PCNA mutants with functional Nhp6B using the plasmids pTK23, 24, 26, 27, 28, and 30. The *NHP6B* gene disrupted by the *LEU2* gene was replaced with a fragment containing the intact *NHP6B* gene followed by the *TRP1* gene (that was amplified by PCR from the plasmid pTK23). Strains expressing PCNA-K164R, PCNA-K127R, PCNA-K127R&K164R, and PCNA-D150E were constructed by replacing the *POL30* gene with fragments containing the intact *NHP6B* followed by *LEU2*, and the PCNA mutant alleles (that were prepared by digestion of pTK26, pTK27, pTK28 and pTK30 by BanII). The mutations were confirmed by sequencing the amplified fragments from genomic DNA.

For testing the effect of PCNA overexpression on sister chromatid recombination, the plasmids pRS303-*GALp* or pRS303-*GALp-POL30-ADH1t* was integrated at the *LEU2* locus in a derivative of DD452 (Kanellis et al., 2003). To construct the sister chromatid recombination tester strains containing PCNA mutant alleles or cell-cycle-regulated *ELG1* alleles, the sister chromatid recombination tester locus (containing two truncated fragments of *HIS3* and the *URA3* gene) was amplified from genomic DNA prepared from a derivative of DD452 and integrated into the *TRP1* locus of cells containing PCNA mutant alleles or RDKY5964 (a parent strain of PCNA mutants or cell-cycle-regulated *ELG1* cells).

To construct strains expressing S-, G2/M-, or M/G1-tagged Elg1, the plasmids pRDK1597, pRDK1598 (Hombauer et al., 2011), or pTK31 were used as template DNAs in PCR reactions. These PCR products targeted the chromosomal *ELG1* locus, leaving the *ELG1* gene under control of the cyclin or a CDK inhibitor promoter and

fusing the N-terminal domains of these cyclins or CDK inhibitor to the N-terminus of Elg1 at amino acid 2.

Fig. S1

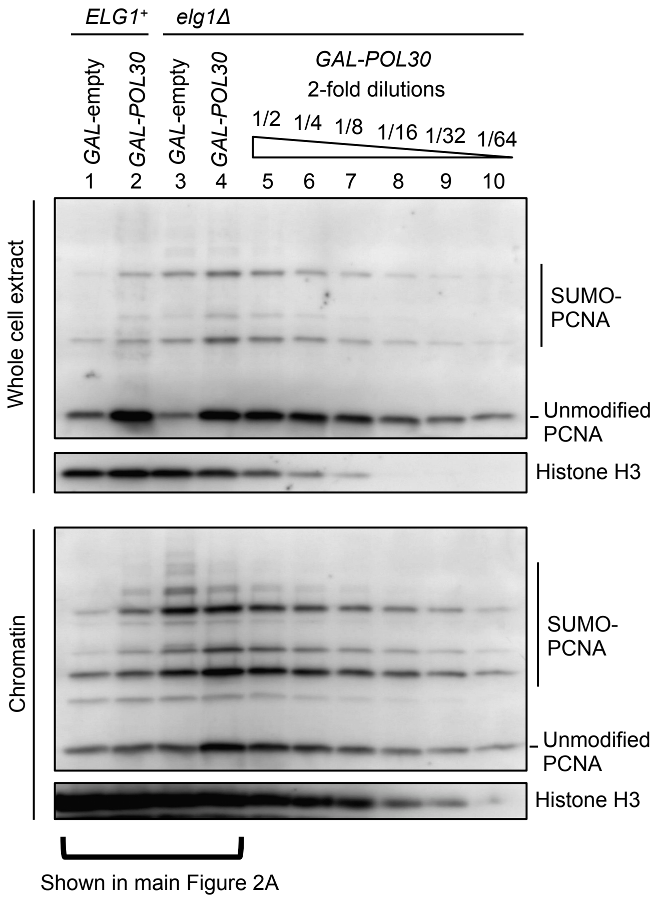
ELG1+
elg1Δ
GAL-empty
GAL-POL30
GAL-empty
GAL-POL30
GAL-POL30
2-fold dilutions
1/2 1/4 1/8 1/16 1/32 1/64
1 2 3 4 5 6 7 8 9 10
Whole cell extract
SUMO-PCNA
Unmodified PCNA
Histone H3
Chromatin
SUMO-PCNA
Unmodified PCNA
Histone H3
Shown in main Figure 2A

Fig. S2

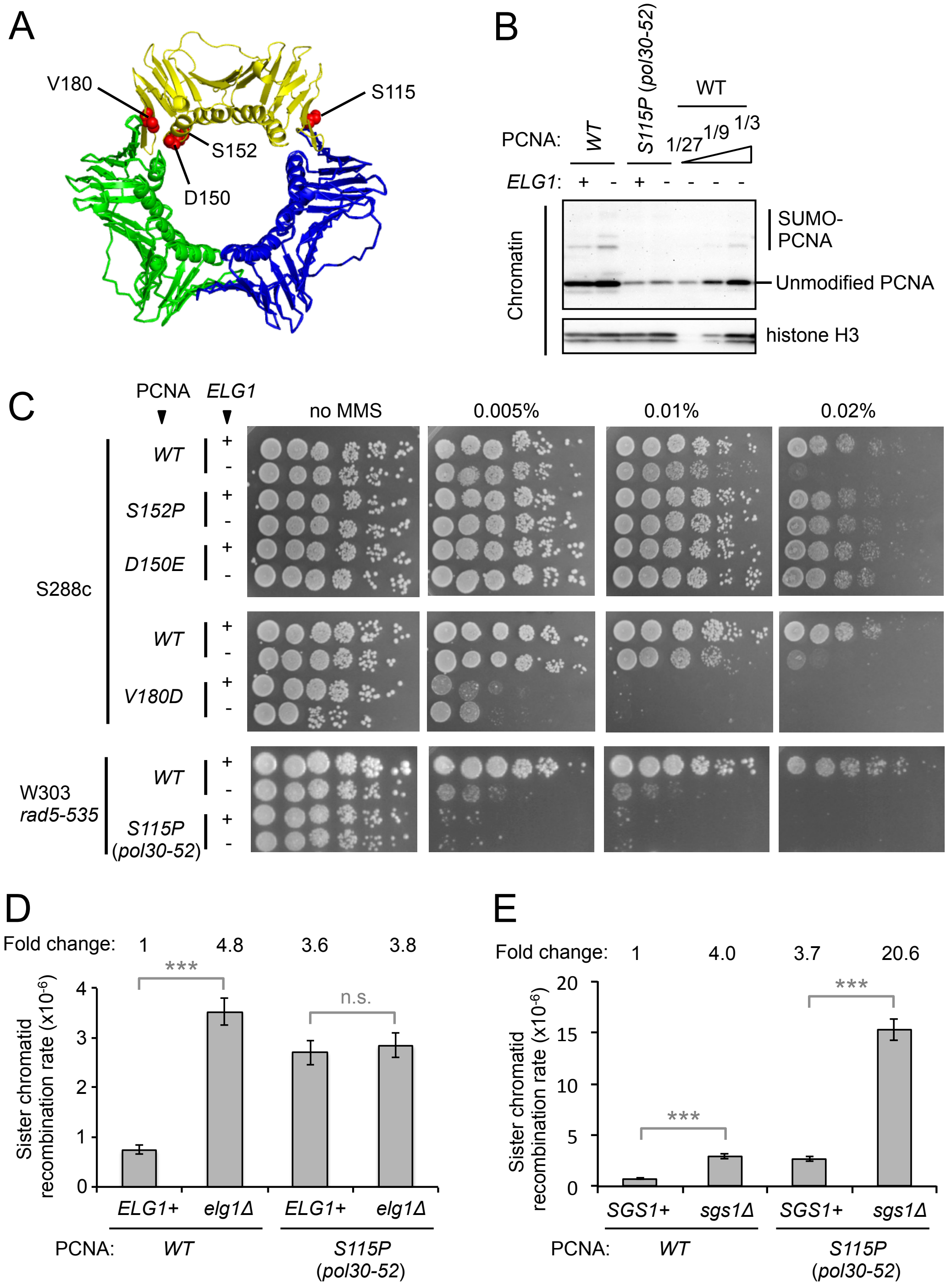
A
V180
S115
S152
D150
B
PCNA:
WT
S115P (pol30-52)
WT
1/27
1/9
1/3
ELG1:
+
-
+
-
-
-
-
Chromatin
SUMO-PCNA
Unmodified PCNA
histone H3
C
PCNA
ELG1
no MMS
0.005%
0.01%
0.02%
S288c
WT
S152P
D150E
WT
V180D
W303 rad5-535
WT
S115P (pol30-52)
D
Fold change: 1 4.8 3.6 3.8
***
n.s.
Sister chromatid recombination rate (x10-6)
4
3
2
1
0
ELG1+
elg1Δ
ELG1+
elg1Δ
PCNA:
WT
S115P (pol30-52)
E
Fold change: 1 4.0 3.7 20.6
***
***
Sister chromatid recombination rate (x10-6)
20
15
10
5
0
SGS1+
sgs1Δ
SGS1+
sgs1Δ
PCNA:
WT
S115P (pol30-52)

Fig. S3

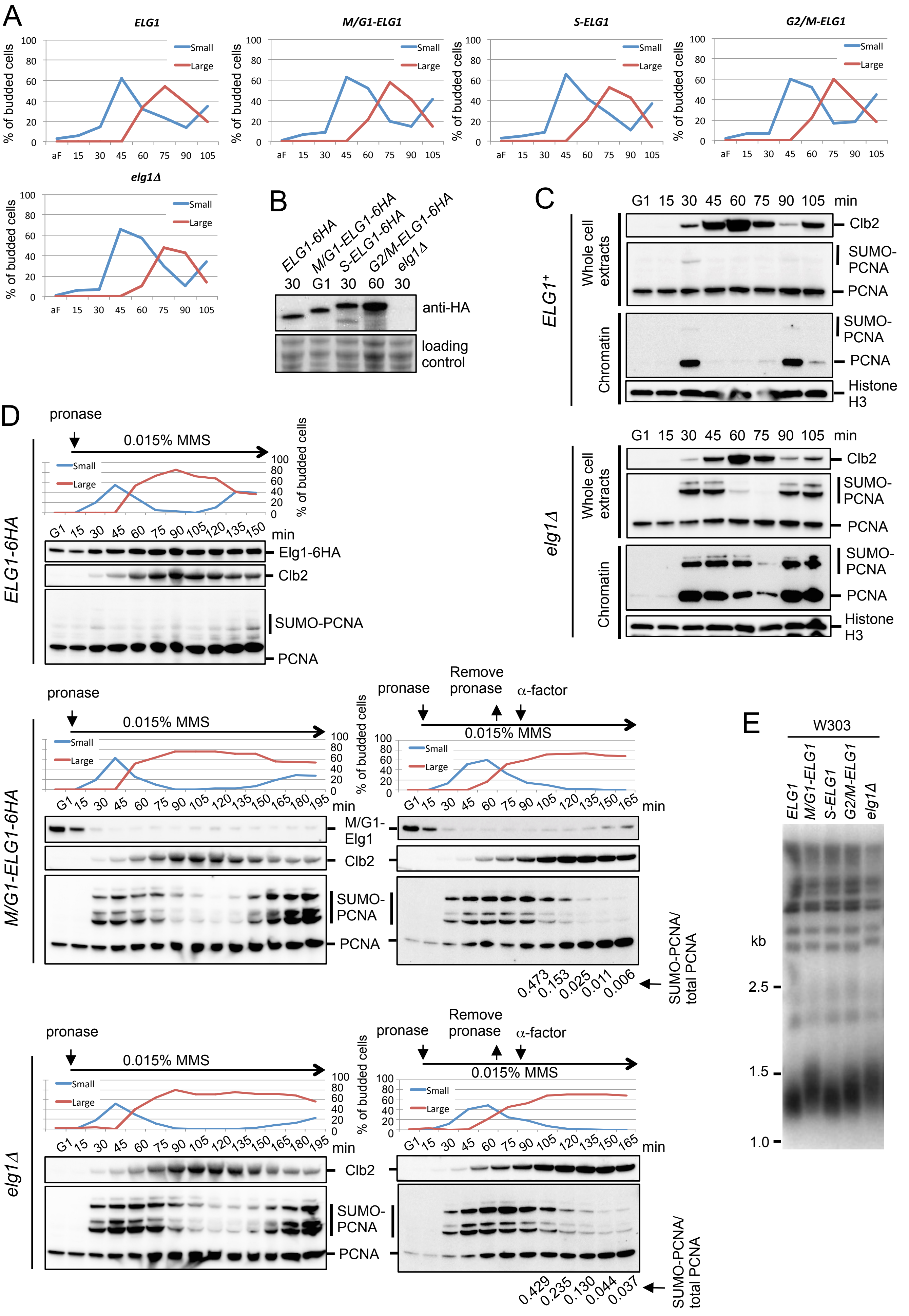
A
ELG1
M/G1-ELG1
S-ELG1
G2/M-ELG1
elg1Δ
% of budded cells
Small
Large
aF 15 30 45 60 75 90 105
B
ELG1-6HA
M/G1-ELG1-6HA
S-ELG1-6HA
G2/M-ELG1-6HA
elg1Δ
30 G1 30 60 30
anti-HA
loading control
C
G1 15 30 45 60 75 90 105 min
ELG1+
elg1Δ
Whole cell extracts
Chromatin
Clb2
SUMO-PCNA
PCNA
Histone H3
D
pronase
0.015% MMS
Remove pronase
α-factor
ELG1-6HA
M/G1-ELG1-6HA
elg1Δ
Elg1-6HA
M/G1-Elg1
Clb2
SUMO-PCNA
PCNA
G1 15 30 45 60 75 90 105 120 135 150 165 180 195 min
SUMO-PCNA/total PCNA
0.473 0.153 0.025 0.011 0.006
0.429 0.235 0.130 0.044 0.037
E
W303
ELG1
M/G1-ELG1
S-ELG1
G2/M-ELG1
elg1Δ
kb
2.5
1.5
1.0

Fig. S4

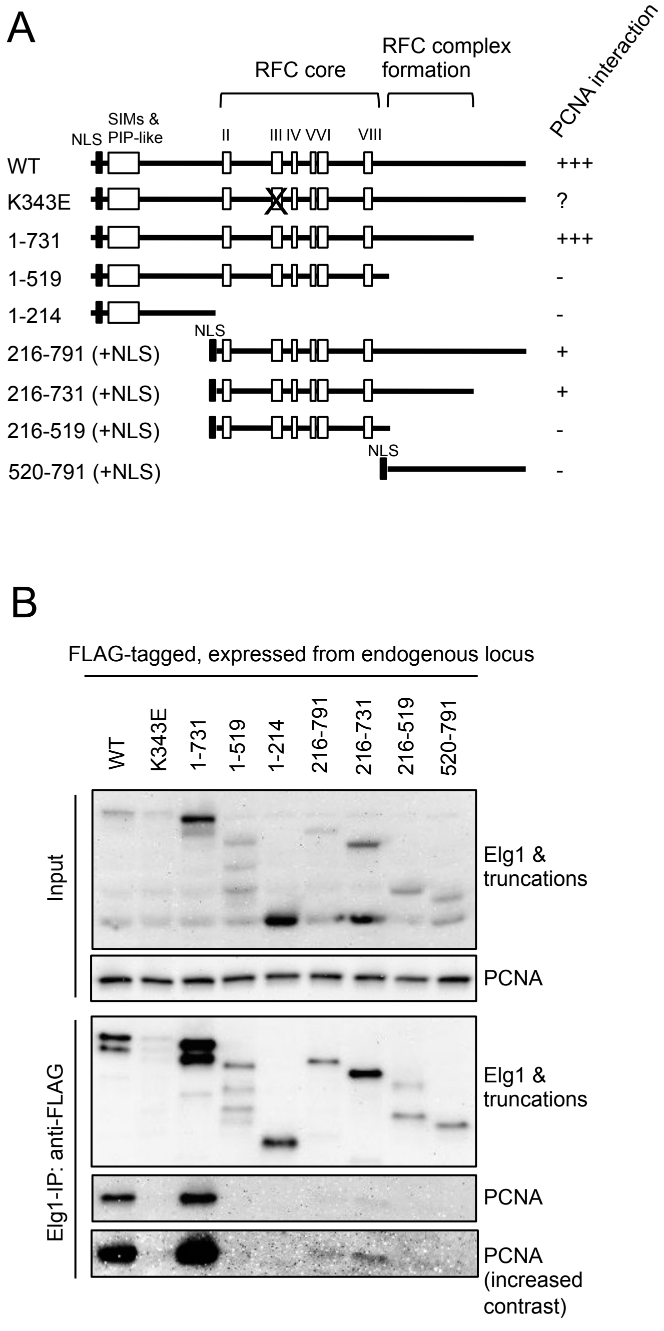
A
RFC core
RFC complex formation
PCNA interaction
NLS
SIMs & PIP-like
II
III IV VVI
VIII
WT +++
K343E ?
1-731 +++
1-519 -
1-214 -
216-791 (+NLS) +
216-731 (+NLS) +
216-519 (+NLS) -
520-791 (+NLS) -
B
FLAG-tagged, expressed from endogenous locus
WT
K343E
1-731
1-519
1-214
216-791
216-731
216-519
520-791
Input
Elg1 & truncations
PCNA
Elg1-IP: anti-FLAG
Elg1 & truncations
PCNA
PCNA (increased contrast)

Supplemental Figure legends

Figure S1, related to main Figure 2. Western blots to quantify PCNA amounts. Whole cell extracts and chromatin-enriched fractions were prepared as described in the Figure legend of main Figure 2. Two-fold serial dilutions of protein samples prepared from *GAL-POL30 elg1Δ* (lanes 5-10) were shown here and used for quantification. For the quantification, the intensity of all PCNA bands in each lane was measured and the intensity of the empty lane (not shown) was subtracted from it. Using 2-fold serial dilutions, relative amounts to *ELG1*$^{+}$ *GAL-empty* were calculated. The values normalised to histone H3 are shown in main Figure 2A.

Figure S2, related to main Figure 3. The trimer interface mutant alleles of PCNA.
(**A**) Structure of the PCNA trimer. The positions mutated are highlighted.
(**B**) The trimer interface PCNA mutant S115P does not accumulate on chromatin in *elg1*Δ. Chromatin-enriched fractions (Chromatin) were prepared from cells expressing wild-type PCNA or the S115P mutant. PCNA and histone H3 (loading control) were detected by western blotting.
(**C**) Deletion of *ELG1* does not sensitise cells to MMS in the trimer interface PCNA mutant background. Five-fold serial dilutions of *ELG1*$^{+}$ or *elg1*Δ expressing wild-type or trimer interface mutant PCNA (S152P, D150E, V180D or S115P) were spotted on YPD medium plus or minus MMS. Cells were incubated for 2-3 days at 30°C.
(**D**) Sister chromatid recombination rate of *ELG1*$^{+}$ and *elg1*Δ in the *S115P* background. *S115P* itself showed increased sister chromatid recombination, but deleting *ELG1* did not increase it further. Error bars, 95% confidence intervals. Mann-Whitney; ***, p-value < 0.0001; n.s., p-value > 0.05.
(**E**) In contrast to *elg1Δ*, sister chromatid recombination rate of *sgs1*Δ was further increased by the trimer interface mutant allele (*S115P*) of PCNA. Error bars, 95% confidence intervals. Mann-Whitney; ***, p-value < 0.0001.

Figure S3, related to main Figure 5. Analysis of cell-cycle-regulated *ELG1* strains.
(**A**) Percentage of small- or large-budded cells in cell-cycle-regulated *ELG1* strains shown in the main Figure 5B.
(**B**) Comparison of expression levels of Elg1-6HA (at 30 min after release) and M/G1-(G1-arrested), S- (30 min), and G2/M-tagged (60 min) Elg1-6HA.
(**C**) The levels of chromatin-bound PCNA correlate with those of SUMO-PCNA in whole cell extracts. Wild-type and *elg1Δ* cells were released from G1 (alpha-factor arrest) into the cell cycle. Samples collected at the indicated time points were kept on ice and 0.1% sodium azide was added. All samples were processed together to prepare whole cell extracts and chromatin fractions.
(**D**) Analysis of *M/G1-ELG1* cells in 0.015% MMS. *WT*, *M/G1-ELG1,* and *elg1Δ* cells were released from G1 into the cell cycle in the presence of 0.015% MMS and whole cell extracts were prepared at the indicated time points (left panels). *M/G1-ELG1* and *elg1Δ* show a slow cell cycle progression in 0.015% MMS. It appears that M/G1-Elg1 can unload PCNA in late M and G1 (120 min) although its expression was not observed clearly, probably due to asynchrony. Reappearance of SUMO-PCNA at the 150-195 min samples indicates that some cells enter the second S phase. To see PCNA unloading by M/G1-Elg1 in the late M and the second G1 phases in the presence of 0.015% MMS more clearly, *M/G1-ELG1* and *elg1Δ* cells were released from G1 into the cell cycle in 0.015% MMS and then arrested in the second G1 phase (right panels). To arrest cells in the second G1 phase, pronase was removed

by washing and resuspending cells in YPD containing 0.015% (after taking the 60 min samples), and then an excess amount of alpha-factor was added to the culture (after taking the 75 min samples). When M/G1-Elg1 was expressed in the late M and the second G1 phases, faster reduction of SUMO-PCNA was observed (compared to *elg1Δ*), indicating that M/G1-Elg1 has the chance to unload PCNA in the presence of MMS. Quantification of SUMO-PCNA/total PCNA at the 105-165 min time points is shown below.
(**E**) Telomere length of the cell-cycle-regulated alleles of *ELG1*. W303 *RAD5*$^{+}$ strains are shown.

Figure S4, related to main Figure 1. Elg1 can interact with PCNA through its central domain, and the interaction is reinforced by its N-terminal domain.
(**A**) Schematic structure of Elg1 and its mutants.
(**B**) Immunoprecipitation assay to determine the domain that can interact with PCNA. Cells expressing FLAG-tagged Elg1 or its truncations from endogenous locus were used. Immunoprecipitation was performed as described previously (Kubota et al. 2011; Kubota et al. 2013) with minor modifications. Briefly, approximately 1 x 10^9 cells were spheroplasted and lysed in 600 μl low-salt buffer (which contains 50 mM potassium acetate). Soluble lysates were prepared by centrifugation following Benzonase treatment and then used for immunoprecipitation with anti-FLAG antibody coupled to Dynabeads Protein G. FLAG-tagged proteins and associated proteins were eluted using 3xFLAG peptides.

**Supplemental Table S1. Plasmids used in this study, related to Figures 1-5**		
**Plasmid name**	**Relevant Genotype**	**Reference**
pRS315	*amp^r CEN6 ARSH4 LEU2*	Sikorski and Hieter 1989
pRS315-*ELG1-13myc*	*amp^r CEN6 ARSH4 LEU2 P_{ELG1}-ELG1-13myc*	Davidson and Brown 2008
pRS315-*elg1(1-731)-13myc*	*amp^r CEN6 ARSH4 LEU2 P_{ELG1}-elg1(1-731)-13myc*	Davidson and Brown 2008
pRS315-*elg1(1-519)-13myc*	*amp^r CEN6 ARSH4 LEU2 P_{ELG1}-elg1(1-519)-13myc*	Davidson and Brown 2008
pRS315-*elg1(1-214)-13myc*	*amp^r CEN6 ARSH4 LEU2 P_{ELG1}-elg1(1-214)-13myc*	Davidson and Brown 2008
pRS315-*elg1(NLS-216-791)-13myc*	*amp^r CEN6 ARSH4 LEU2 P_{ELG1}-elg1(NLS-216-791)-13myc*	Davidson and Brown 2008
pRS315-*elg1(NLS-216-731)-13myc*	*amp^r CEN6 ARSH4 LEU2 P_{ELG1}-elg1(NLS-216-731)-13myc*	This study
pRS315-*elg1(NLS-216-519)-13myc*	*amp^r CEN6 ARSH4 LEU2 P_{ELG1}-elg1(NLS-216-519)-13myc*	Davidson and Brown 2008
pRS315-*elg1(NLS-520-791)-13myc*	*amp^r CEN6 ARSH4 LEU2 P_{ELG1}-elg1(NLS-520-791)-13myc*	This study
pTK23	*amp^r NHP6B TRP1 P_{POL30}-POL30*	This study
pTK24	*amp^r NHP6B LEU2 P_{POL30}-POL30*	This study
pTK26	*amp^r NHP6B LEU2 P_{POL30}- pol30-K164R*	This study
pTK27	*amp^r NHP6B LEU2 P_{POL30}-pol30-K127R*	This study
pTK28	*amp^r NHP6B LEU2 P_{POL30}-pol30-K127R&K164R*	This study
pTK30	*amp^r NHP6B LEU2 P_{POL30}-pol30-D150E*	This study
pTK31	*amp^r natNT2 P_{SIC1}-sic1(1-105aa)*	This study
pRDK1597	*amp^r natNT2 P_{CLB6}-clb6(1-195aa)*	Hombauer et al. 2011
pRDK1598	*amp^r natNT2 P_{CLB2}-clb2(1-181aa, L26A)*	Hombauer et al. 2011
pRS303-*GALp*	*amp^r LEU2 $P_{GAL1-10}$*	This study
pRS303-*GALp-POL30-ADH1t*	*amp^r LEU2 $P_{GAL1-10}$-POL30-T_{ADH1}*	This study
