# Supplementary material for: PCNA Retention on DNA into G2/M Phase Causes Genome Instability in Cells Lacking Elg1
Source: Cell Rep. 2016 Jun 30;16(3):684–95. doi: 10.1016/j.celrep.2016.06.030 (PMC4956615; doi:10.1016/j.celrep.2016.06.030)
Supplement: Document S2. Article plus Supplemental Information [file mmc3.pdf]

Cell Reports

Article

PCNA Retention on DNA into G2/M Phase Causes Genome Instability in Cells Lacking Elg1

Graphical Abstract

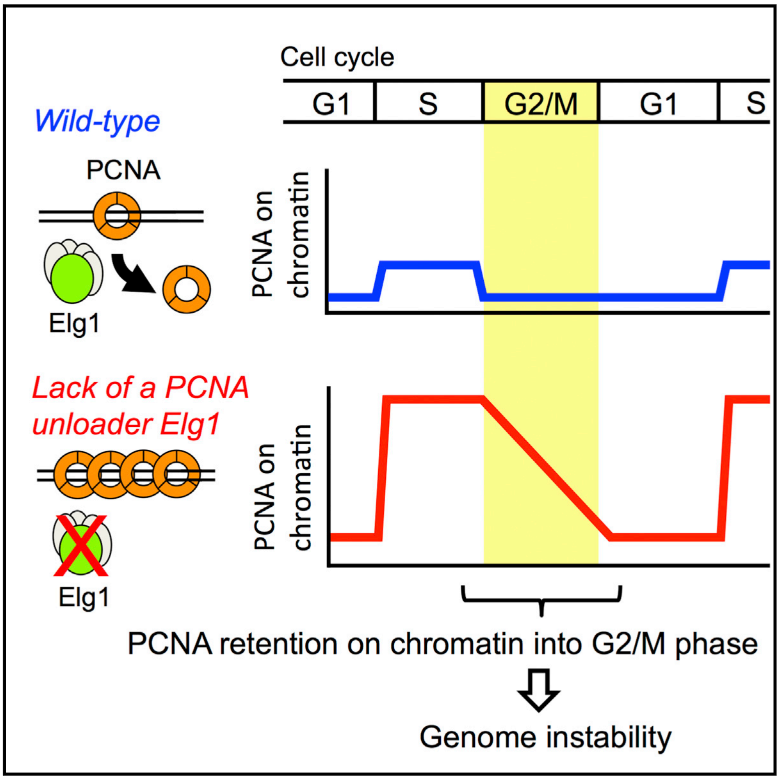

Authors

Catherine Johnson, Vamsi K. Gali, Tatsuro S. Takahashi, Takashi Kubota

Correspondence

t.kubota@abdn.ac.uk

In Brief

Loss of the genome maintenance factor Elg1 causes serious genome instability. Johnson et al. find that PCNA unloading by Elg1 is critical for genome maintenance, and that PCNA retention on DNA into G2/M phase causes genome instability in cells lacking Elg1.

Highlights

- PCNA accumulation on DNA causes genome instability
- Relieving PCNA accumulation on DNA rescues genome instability in *elg1Δ* cells
- PCNA retention on DNA into G2/M phase causes genome instability in cells lacking Elg1
- PCNA unloading by Elg1-RLC is critical for genome maintenance

Johnson et al., 2016, Cell Reports *16*, 684–695
July 19, 2016 © 2016 The Author(s).
http://dx.doi.org/10.1016/j.celrep.2016.06.030

OPEN ACCESS
CellPress

Cell Reports
Article

PCNA Retention on DNA into G2/M Phase Causes Genome Instability in Cells Lacking Elg1

Catherine Johnson,[1] Vamsi K. Gali,[1] Tatsuro S. Takahashi,[2] and Takashi Kubota[1,*]
[1]Institute of Medical Sciences, University of Aberdeen, Foresterhill, Aberdeen AB25 2ZD, Scotland, UK
[2]Graduate School of Science, Osaka University, 1-1 Machikaneyama-cho, Toyonaka, Osaka 560-0043, Japan
*Correspondence: t.kubota@abdn.ac.uk
http://dx.doi.org/10.1016/j.celrep.2016.06.030

SUMMARY

Loss of the genome maintenance factor Elg1 causes serious genome instability that leads to cancer, but the underlying mechanism is unknown. Elg1 forms the major subunit of a replication factor C-like complex, Elg1-RLC, which unloads the ring-shaped polymerase clamp PCNA from DNA during replication. Here, we show that prolonged retention of PCNA on DNA into G2/M phase is the major cause of genome instability in *elg1Δ* yeast. Overexpression-induced accumulation of PCNA on DNA causes genome instability. Conversely, disassembly-prone PCNA mutants that relieve PCNA accumulation rescue the genome instability of *elg1Δ* cells. Covalent modifications to the retained PCNA make only a minor contribution to *elg1Δ* genome instability. By engineering cell-cycle-regulated *ELG1* alleles, we show that abnormal accumulation of PCNA on DNA during S phase causes moderate genome instability and its retention through G2/M phase exacerbates genome instability. Our results reveal that PCNA unloading by Elg1-RLC is critical for genome maintenance.

INTRODUCTION

Maintenance of the genome is crucial for all living organisms since loss of genome stability leads to mutations and chromosome rearrangements, causing cancers and other life-threatening diseases (Aguilera and Gómez-González, 2008; Negrini et al., 2010). Cells deploy multiple mechanisms to prevent genome instability, including error-free replication of the genome in S phase, efficient repair of DNA damage, and faithful transmission of the genome to daughter cells. Loss of factors involved in these processes generally causes profound genome instability (Mailand et al., 2013; Negrini et al., 2010).

Elg1, the major subunit of a replication factor C-like complex, is critical for genome maintenance. In budding yeast, loss of the *ELG1* gene causes gross chromosomal rearrangements, increased sister chromatid recombination, defective sister chromatid cohesion, derailed telomere length maintenance, and sensitivity to the DNA alkylating drug methyl methanesulfonate (MMS) (Banerjee and Myung, 2004; Bellaoui et al., 2003; Ben-Aroya et al., 2003; Kanellis et al., 2003; Maradeo and Skibbens, 2009; Parnas et al., 2009; Smolikov et al., 2004). The requirement for Elg1 in genome maintenance is conserved in higher eukaryotes, since mice with reduced expression of ATAD5 (the mammalian ortholog of Elg1) exhibit genome instability and have a high tumor incidence (Bell et al., 2011). In humans, somatic mutations in ATAD5 have been found in primary endometrial tumors (Bell et al., 2011). ATAD5 was moreover recently identified as a susceptibility locus for invasive epithelial ovarian cancer (Kuchenbaecker et al., 2015). Despite its evident importance, the mechanism by which Elg1 ensures genome integrity is unknown.

One molecular function of yeast Elg1 Replication factor C-like complex (Elg1-RLC) is to unload the proliferating cell nuclear antigen (PCNA) sliding clamp from DNA during replication (Kubota et al., 2013b; Ulrich, 2013). PCNA has a central role in DNA replication, repair, and chromatin dynamics, as illustrated by a mutation in human PCNA associated with DNA repair deficiency syndrome akin to diseases like xeroderma pigmentosum, Cockayne syndrome, and ataxia telangiectasia (Baple et al., 2014; Duffy et al., 2016). PCNA is a ring-shaped homotrimeric complex that encircles DNA to act as a sliding clamp, ensuring processivity of DNA polymerases. It also operates as a platform for recruitment of numerous other proteins involved in DNA replication, DNA repair and chromatin structure and assembly (Moldovan et al., 2007). During DNA replication, Replication Factor C (RFC) must load PCNA at the initiation of synthesis of each Okazaki fragment. The hetero-pentameric RFC complex, composed of largest subunit Rfc1 and smaller subunits Rfc2, 3, 4, and 5, loads PCNA at primer-template junctions (Bowman et al., 2004; Gomes and Burgers, 2001; Kelch et al., 2011). After completion of each Okazaki fragment, PCNA must be unloaded from DNA and is believed to be recycled to promote subsequent Okazaki fragment synthesis. Previous findings indicate that the Elg1-RLC, which comprises the Elg1 subunit associated with the Rfc2–5 subunits, functions to unload PCNA during replication (Kubota et al., 2013a, 2013b). This replication-coupled PCNA unloading by Elg1-RLC occurs genome-wide (as opposed to at specific loci) and requires prior Okazaki fragment ligation (Kubota et al., 2015). The role of the Elg1-RLC in PCNA unloading appears to be conserved in humans, since ATAD5 is required for proper removal of PCNA from chromatin in human cell lines (Lee et al., 2013; Shiomi and Nishitani, 2013).

PCNA can be modified by ubiquitin and small ubiquitin-related modifier (SUMO), modulating its physical interactions with various binding partners. PCNA ubiquitination at K164 is induced

 © 2016 The Author(s).
This is an open access article under the CC BY license (http://creativecommons.org/licenses/by/4.0/).

by replication stress associated with fork stalling (Davies et al., 2008; Hoege et al., 2002). K164 mono-ubiquitinated PCNA mediates an error-prone DNA damage tolerance pathway by recruiting translesion synthesis polymerases that can replicate past a DNA lesion (Bienko et al., 2005; Stelter and Ulrich, 2003). K164 poly-ubiquitinated PCNA in contrast mediates an error-free mode of damage bypass that involves template switch recombination using the sister chromatid (Hoege et al., 2002; Parker and Ulrich, 2009; Stelter and Ulrich, 2003). In budding yeast, SUMOylation of PCNA at K164 and K127 is stimulated simply by DNA association and occurs during S phase without exogenous DNA damage (Parker et al., 2008). One role for PCNA SUMOylation is to prevent inappropriate recombination through recruitment of DNA helicase Srs2 that prevents formation of Rad51 filaments (Papouli et al., 2005; Pfander et al., 2005). Budding yeast Elg1-RLC preferentially interacts with SUMOylated PCNA through three SUMO-interacting motifs (SIMs) (Parnas et al., 2010). SUMOylation may assist but is not necessary for PCNA unloading (Kubota et al., 2013b), and the importance of the SUMO binding activity of Elg1 for PCNA unloading is not yet fully understood.

The Elg1-RLC is thought to function in processes other than PCNA unloading. Yeast Elg1 can interact with several additional proteins—including SUMOylated proteins, SUMO-processing proteins, and the SUMO-like domain protein Esc2—mediated by the N-terminal region of Elg1 which contains the SIMs (Parnas et al., 2011; Urulangodi et al., 2015).

It is unknown whether the dramatic genome instability that occurs in the absence of Elg1 is caused by failure of PCNA unloading, or instead by the loss of interaction with other Elg1 binding partners. Here, we address this issue, and our results reveal that aberrant retention and accumulation of PCNA on DNA is the major cause of genome instability in *elg1*Δ. We show that overexpression of PCNA causes its accumulation on DNA, resulting in genome instability resembling that caused by the *elg1*Δ mutation. Relieving PCNA accumulation through the use of disassembly-prone PCNA mutants results in rescue of all aspects of the *elg1*Δ phenotype tested. To assess whether PCNA retention is deleterious at a particular cell-cycle stage, we construct cell-cycle-regulated alleles of *ELG1*. Analysis of their impact shows that abnormal PCNA retention on DNA beyond DNA replication and into G2/M phase is the root cause of genome instability.

RESULTS

ELG1 Truncation Mutants Causing PCNA Accumulation on Chromatin Exhibit Increased Sensitivity to MMS

Impaired Elg1 function causes sensitivity to MMS (Davidson and Brown, 2008). We first tested whether there is a correlation between MMS sensitivity and failure of Elg1 to unload PCNA. Either full-length or various mutant forms of Elg1 were expressed from plasmids in an *elg1*Δ background (Davidson and Brown, 2008) (Figure 1A). Their ability to unload PCNA was quantified by assessing PCNA present in chromatin fractions and using western blots of whole-cell extracts (where SUMO-PCNA acts as a proxy for the PCNA pool that is chromatin bound). As expected, Elg1 truncations unable to form a complex with other RFC subunits (Davidson and Brown, 2008) were generally defective in unloading PCNA from chromatin (Figure 1B). Interestingly, the levels of PCNA accumulation on chromatin caused by these *ELG1* truncation mutants largely correlate with increased sensitivity to MMS (Figures 1B and 1C). In particular, the N-terminal domain of Elg1, which contains the SUMO-interacting motifs (SIMs), is not essential for MMS resistance and PCNA unloading (Figures 1B and 1C, 216–791)—despite its importance for mediating interaction with SUMOylated proteins (Parnas et al., 2011). This observation that MMS sensitivity generally mirrors failure of PCNA unloading led us to design further experiments aimed at examining whether PCNA hyper-accumulation on DNA is in fact the major cause of the MMS sensitivity and other defects observed in an *elg1*Δ mutant.

Overexpression of PCNA Causes Its Accumulation on Chromatin and Exacerbates Phenotypes of *elg1*Δ

We envisaged two general mechanisms through which failed PCNA unloading in the absence of Elg1 might cause genome instability. Genome instability might be due to PCNA accumulation on DNA that interferes with subsequent chromosome transactions, or else due to a shortage of PCNA at sites of DNA synthesis caused by delayed recycling of PCNA as a consequence of failed unloading. Partly to differentiate between these models, we examined effects of overexpressing PCNA. In the latter case, genome instability of *elg1*Δ would be rescued by supplying excess PCNA during overexpression. We constructed strains where the *POL30* gene (encoding PCNA in budding yeast) is fused to a galactose-inducible promoter (*GAL-POL30*) and integrated in the genome, so that PCNA overexpression can be induced by galactose addition. Even in the presence of the PCNA unloader Elg1, overexpressing PCNA caused some accumulation on DNA (Figure 2A, lanes 1 and 2; Figure S1). We also found that overexpressing PCNA in an *ELG1*$^{+}$ background sensitizes cells to MMS (Figure 2B), consistent with excess PCNA on chromatin causing the increased MMS sensitivity. Supporting this idea, overexpressing PCNA in the absence of Elg1 results in hyper-accumulation of PCNA on chromatin (Figure 2A, lanes 3 and 4; Figure S1), and extreme sensitivity to MMS (Figure 2B). We also observed that, even without MMS, growth of the *elg1*Δ mutant strains is impaired when PCNA is overexpressed (Figure 2B, no MMS, Gal). These results support the idea that increased sensitivity to MMS of *elg1*Δ is caused by PCNA accumulation on chromatin, rather than by a shortage of PCNA at replication forks.

Loss of the PCNA unloader Elg1 causes elevated levels of sister chromatid recombination, indicative of genome instability (Kanellis et al., 2003). We next tested whether PCNA accumulation on DNA induced by its overexpression causes increased sister chromatid recombination. We used strains in which two tandem fragments of the *HIS3* gene are integrated upstream of the *TRP1* locus; one fragment lacks the 5′ region (*his3Δ5′*) and the other lacks the 3′ region (*his3Δ3′*), with each fragment containing around 300 bp of identical sequence in the central region of the gene (Fasullo and Davis, 1987) (Figure 2B, top). These strains lack endogenous *HIS3*, so that a functional *HIS3* gene can only be generated via inter-sister-chromatid recombination. We observed that PCNA overexpression caused a slight increase of spontaneous sister chromatid recombination rate in the presence of Elg1, which was greatly exacerbated in the absence

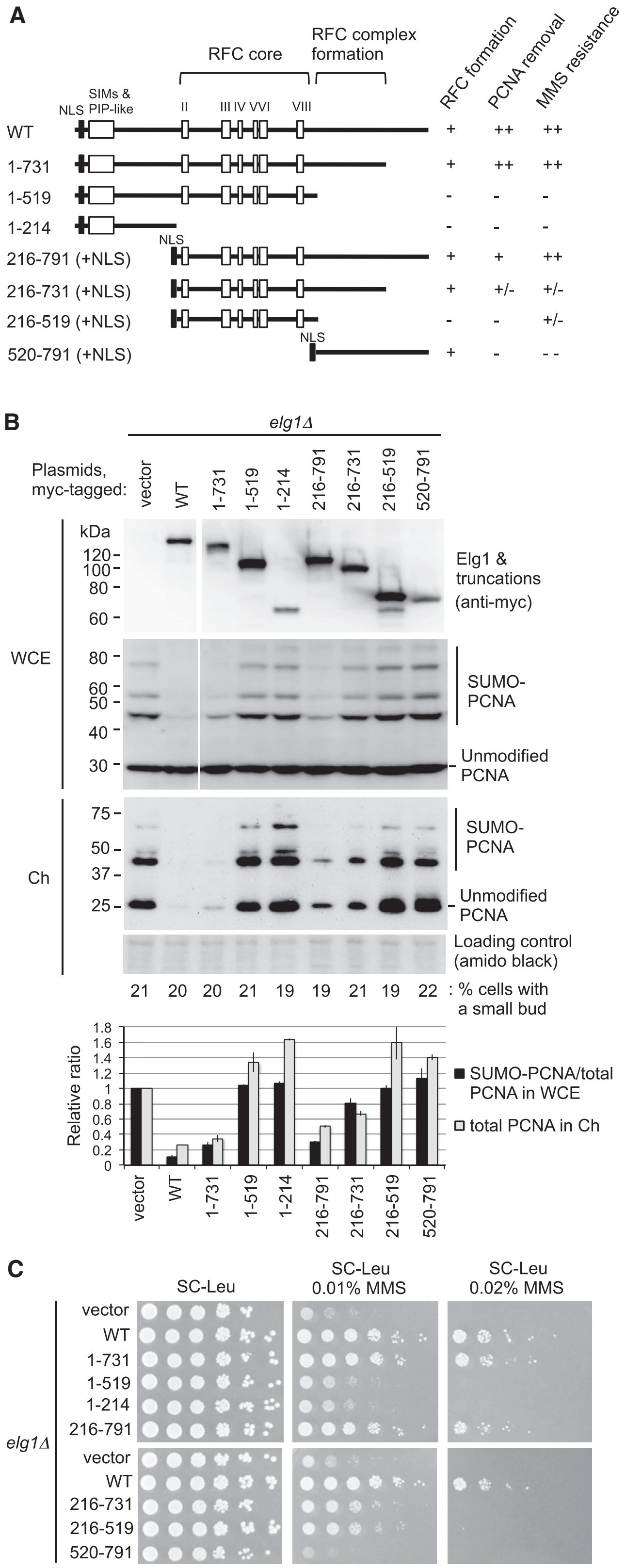

Figure 1. Cells Showing PCNA Accumulation on Chromatin Caused by Truncation of *ELG1* Exhibit Increased Sensitivity to MMS

(A) Schematic structure of Elg1 and truncated mutants. Interaction of truncated Elg1 with Rfc4 (RFC formation) was examined previously (Davidson and Brown, 2008). NLS, nuclear localization signal; SIMs, SUMO interacting motifs; PIP-like, PCNA interacting peptide-like motif.

(B) Accumulation of SUMO-PCNA in whole-cell extracts and PCNA on chromatin in cells expressing truncated Elg1 fragments. Whole-cell extracts (WCE) and chromatin-enriched fractions (Ch) were prepared from *elg1*Δ cells, carrying the empty plasmid or plasmids containing truncated alleles of *ELG1*, in log phase. Truncated Elg1 and PCNA were detected by western blotting. Percentage of cells with small buds, indicative of cells in S phase, is shown below blots. Quantification of average of two experiments was shown. Error bars, SDs.

(C) Sensitivity to MMS of *elg1*Δ cells carrying the empty plasmid or plasmids containing truncated alleles of *ELG1*. 5-fold serial dilutions of cells were spotted on synthetic medium lacking leucine with 2% glucose, plus or minus MMS, and incubated for 3–4 days at 30°C.

See also Figure S4.

of Elg1 (Figure 2C, bottom). These results are consistent with the idea that as with MMS sensitivity, increased sister chromatid recombination in *elg1*Δ is caused by PCNA accumulation on DNA.

Disassembly-Prone PCNA Mutants Rescue MMS Sensitivity of *elg1*Δ

If the phenotypes of *elg1*Δ are caused by PCNA accumulation, then removal of PCNA from DNA should rescue the phenotypes. To relieve the accumulation of PCNA on DNA caused by *ELG1* deletion, we utilized PCNA mutants that in vitro cannot form stable trimers due to point mutations at the trimer interface (C81R, E143K, and D150E; Figure 3A, red) (Dieckman et al., 2013; Goellner et al., 2014; Lau et al., 2002). We first tested the effects of these trimer interface mutants in vivo and found that all three mutations prevent accumulation of PCNA on chromatin in the absence of Elg1 (Figure 3B), consistent with their spontaneous dissociation from DNA due to trimer instability. Although their markedly reduced chromatin association indicates that these PCNA mutants dissociate prematurely from DNA, cells expressing the mutants show no obvious growth defect at 30°C in the absence of MMS (Figure 3C, no MMS plates) (see Discussion).

We then examined whether disassembly-prone PCNA mutants rescue the sensitivity of *elg1*Δ to MMS. Remarkably, the increased MMS sensitivity caused by the *elg1*Δ mutation was rescued by the disassembly-prone PCNA alleles *E143K* and *D150E* (Figure 3C). Although the disassembly-prone C81R mutation itself sensitized cells to MMS, deletion of *ELG1* in the C81R mutant likewise did not cause further MMS sensitivity (Figure 3C). In fact, this characteristic is shared by all trimer interface mutants tested (Figures S2A–S2C), including S152P, V180D, and the S115P mutant (also known as *pol30–52*) (Ayyagari et al., 1995; Goellner et al., 2014). Among these, the lack of accumulation of the S115P mutant on chromatin has been confirmed (Figure S2B). These results strongly suggest that the MMS sensitivity associated with loss of Elg1 is a consequence of hyper-accumulation of PCNA on DNA. The differential MMS sensitivity of individual disassembly-prone PCNA mutants is likely due to differences in the nature of the mutations and how they affect trimer stability or recruitment of additional factors by PCNA.

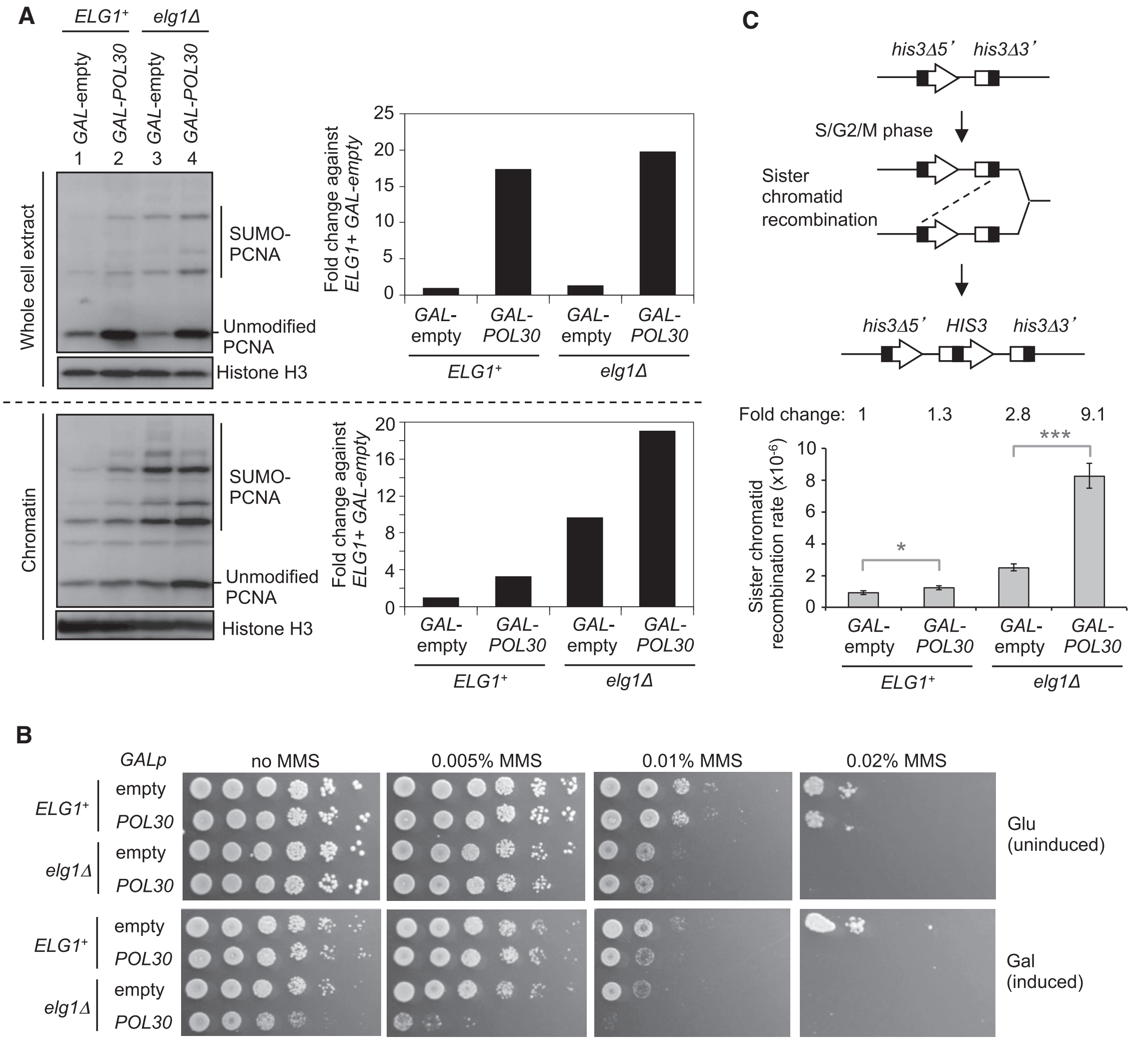

Figure 2. Overexpression of PCNA Causes Its Accumulation on Chromatin and Exacerbates Phenotypes of *elg1Δ*

(A) Overexpression of PCNA causes its accumulation on chromatin in *ELG1*$^+$ and exacerbates PCNA accumulation on chromatin in *elg1*Δ. Cells were arrested in G1 phase by alpha-factor, and PCNA overexpression was induced for 2 hr by adding galactose prior to releasing into S phase. Cells were then collected at mid-S phase (35 min after release) and whole-cell extract and chromatin-enriched fractions were prepared. PCNA levels were examined by western blotting (left panels). Quantitation of total PCNA including both unmodified and modified PCNA (normalized to histone H3) is shown, relative to *ELG1*$^+$ with empty vector (right panels and see also Figure S1). The *POL30* gene encodes PCNA.

(B) Overexpression of PCNA sensitizes *ELG1*$^+$ and *elg1*Δ cells to MMS and causes slow growth of *elg1*Δ. 5-fold serial dilutions of *ELG1*$^+$ or *elg1*Δ carrying the *GALp* plasmid (empty) or *GALp-POL30* plasmid (*POL30*) integrated in the genome were spotted on synthetic medium (without histidine) with 2% glucose (Glu; uninduced) or 2% galactose + 2% raffinose (Gal; induced), plus or minus MMS. Plates were incubated for 3–4 days at 30°C.

(C) Spontaneous sister chromatid recombination assay. The sister chromatid recombination substrate (top) is composed of one 5′ and one 3′ deletion fragment of the *HIS3* gene, inserted at the *TRP1* locus. Formation of a functional *HIS3* gene only occurs via sister chromatid recombination (either reciprocal recombination or gene conversion) between the *HIS3* deletion fragments that possess a region of overlap (black region). Spontaneous sister chromatid recombination rate (bottom) was calculated as described in Experimental Procedures. Fold increase over wild-type is shown above. Error bars, 95% confidence intervals. Mann-Whitney; ***p value <0.0001; *p value <0.05.

See also Figure S1.

Disassembly-Prone PCNA Mutants Rescue the Increased Sister Chromatid Recombination and Abnormal Elongation of Telomeres of *elg1Δ*

We next tested whether the increased sister chromatid recombination and abnormal elongation of telomeres observed in *elg1*Δ are rescued by relieving accumulation of PCNA on DNA. Deletion of *ELG1* caused an ~3-fold increase in spontaneous sister chromatid recombination rate at the tester locus (as illustrated in Figure 2C) that was largely rescued by the disassembly-prone PCNA mutants D150E, E143K, and C81R (Figure 3D). PCNA mutant D150E could not, however, rescue elevated sister chromatid recombination rate caused by absence of Sgs1, a RecQ family DNA helicase required for processing recombination intermediates (Ashton and Hickson,

2010; Kanellis et al., 2003) (Figure 3E). This control confirms that sister chromatid recombination can still occur in a trimer instability mutant and indicates that it is indeed PCNA accumulation on DNA that causes the elevated recombination levels seen in *elg1*Δ.

Deletion of *ELG1* results in telomere elongation that depends on active telomerase (Smolikov et al., 2004). As with the increased sister chromatid recombination, abnormal elongation of telomeres in *elg1*Δ is rescued by disassembly-prone PCNA mutants (Figure 3F). This observation indicates that PCNA accumulation on DNA also deregulates telomere maintenance, through a pathway as yet unknown.

Preventing PCNA Modification in *elg1*Δ Does Not Rescue MMS Sensitivity or Increase Sister Chromatid Recombination, but Partly Rescues Telomere Elongation

PCNA modifications are believed to regulate cellular choice of pathway for DNA repair or damage tolerance (Andersen et al., 2008). We considered the possibility that hyper-accumulation of the SUMOylated PCNA, as opposed to simply PCNA retention, could be the reason why *elg1*Δ cells show increased MMS sensitivity. To address this issue, we tested whether preventing PCNA SUMOylation rescues MMS sensitivity of *elg1*Δ. We used a PCNA K127R mutant and simultaneously deleted the SUMO E3 ligase *SIZ1* whose product SUMOylates PCNA at K164 (Parker et al., 2008). However, loss of SUMOylation of PCNA (in this *siz1*Δ *pol30-K127R* strain) did not rescue the MMS sensitivity of *elg1*Δ (Figure 4A). Preventing both SUMOylation and ubiquitination of PCNA (using a *pol30-K127R&K164R* allele) also failed to rescue the MMS sensitivity of *elg1*Δ (Figure 4A). Note that such unmodifiable PCNA mutants do still over-accumulate on DNA in *elg1*Δ (Kubota et al., 2013b). These results indicate that the MMS sensitivity of *elg1*Δ can result from accumulation of unmodified PCNA on DNA and is not simply caused by over-recruitment of factors recognizing a modified PCNA form.

Likewise, preventing PCNA modification did not rescue the 3-fold increase of sister chromatid recombination seen in *elg1*Δ (Figure 4B), indicating that accumulation of unmodified PCNA is sufficient to cause increased sister chromatid recombination in *elg1*Δ.

We found that preventing PCNA modification can in contrast partly rescue the abnormal telomere elongation in *elg1*Δ. Specifically, in an *elg1*Δ background mutating K127, K164, or both residues to non-modifiable arginine restores telomeres back closer to normal length (Figure 4C). In contrast to the MMS sensitivity and sister chromatid recombination, PCNA modification therefore appears to play a minor role in mediating elongation of telomeres in *elg1*Δ.

Establishing Cell-Cycle-Regulated Alleles that Restrict Elg1 Activity to M/G1, S, or G2/M Phase

In the absence of Elg1, PCNA accumulates on DNA during S phase and lingers abnormally on DNA at subsequent cell-cycle stages (Kubota et al., 2013b). We considered whether it is critical for genome stability that PCNA is removed from DNA by a particular cell-cycle stage. To investigate this possibility, we constructed cell-cycle-regulated alleles that restrict the presence of Elg1 to M/G1, S, or G2/M phase (Figure 5A) by taking advantages of cell-cycle-regulated transcription and protein degradation. Endogenous Elg1 protein is present throughout the cell cycle (Figure 5Bi), and while it normally acts during S phase it can still unload PCNA in G2 phase, after replication is complete (Kubota et al., 2013b). To limit Elg1 activity to late M/G1, the promoter and N-terminal degron domain of the CDK inhibitor Sic1 (hereafter designated the "M/G1-tag") was integrated upstream of the *ELG1* gene, resulting in an *M/G1-ELG1* fusion that expresses only in late M/G1 phase and whose product is degraded in S phase (through phosphorylation-dependent protein degradation [Sheaff and Roberts, 1996]). As expected, M/G1-Elg1-6HA was detected mainly in M/G1 phase (Figure 5Bii, cell-cycle progression and stage confirmed by times of bud emergence and Clb2 expression; Figures S3A and 5B). Similarly, to limit the Elg1 protein to S phase, the promoter and N-terminal degron element of the S phase cyclin Clb6 ("S-tag") (Hombauer et al., 2011) was fused to the *ELG1* gene. Expression of full-length S-Elg1-6HA is largely limited to S phase, although a weaker band, probably corresponding to a degradation product that lacks the S-tag, was detected throughout the cell cycle (Figure 5Biii). Finally, to limit Elg1 to G2/M phase, the promoter and N-terminal region of the mitotic cyclin Clb2 including its degrons ("G2/M-tag") (Hombauer et al., 2011; Karras and Jentsch, 2010) was integrated upstream of *ELG1*. G2/M-Elg1-6HA protein was detected mainly in G2/M phase (Figure 5Biv), concurrent with expression of endogenous Clb2. Although expressed from different promoters, peak expression levels of M/G1-, S-, and G2/M-Elg1 proteins were similar to that of endogenous Elg1 (Figure S3B).

We then quantified PCNA unloading when Elg1 is expressed at defined stages of the cell cycle. In these experiments, levels of SUMOylated PCNA in whole-cell extracts were used to assess PCNA accumulation on DNA (since SUMOylation reflects chromatin-bound PCNA (Parker et al., 2008; Figures 1 and S3C). In wild-type cells, SUMO-PCNA was detected transiently in S phase (Figures 5Bi and 5C), demonstrating that PCNA is loaded during replication and unloaded once replication is finished. In *S-ELG1* cells, we observed a similar pattern of SUMO-PCNA appearance to that in wild-type cells although the level of SUMO-PCNA in *S-ELG1* cells is slightly increased (Figures 5Biii and 5C), suggesting that S-tagged Elg1 can unload PCNA from DNA during replication, but its activity is slightly impaired by the tagging. In the complete absence of Elg1, SUMO-PCNA massively over-accumulated in S phase, and, although levels gradually decreased, they still remained abnormally high as cells entered the next S phase (Figures 5Bv and 5C), indicating that PCNA is retained on DNA even through G2/M phase. PCNA retention on DNA through G2/M phase was confirmed by western blot analysis of chromatin-enriched fractions (Figure S3C). *M/G1-ELG1* cells have an abnormal SUMO-PCNA pattern resembling that of the *elg1*Δ mutant, with PCNA retained on DNA well into G2/M phase. However *M/G1-ELG1* cells showed less SUMO-PCNA than *elg1*Δ at the 75 min time point (cf. Figures 5Bii with 5Bv, and dashed red with solid blue line in Figure 5C)—consistent with the suggestion that M/G1-Elg1 can unload residual PCNA from DNA in late M or

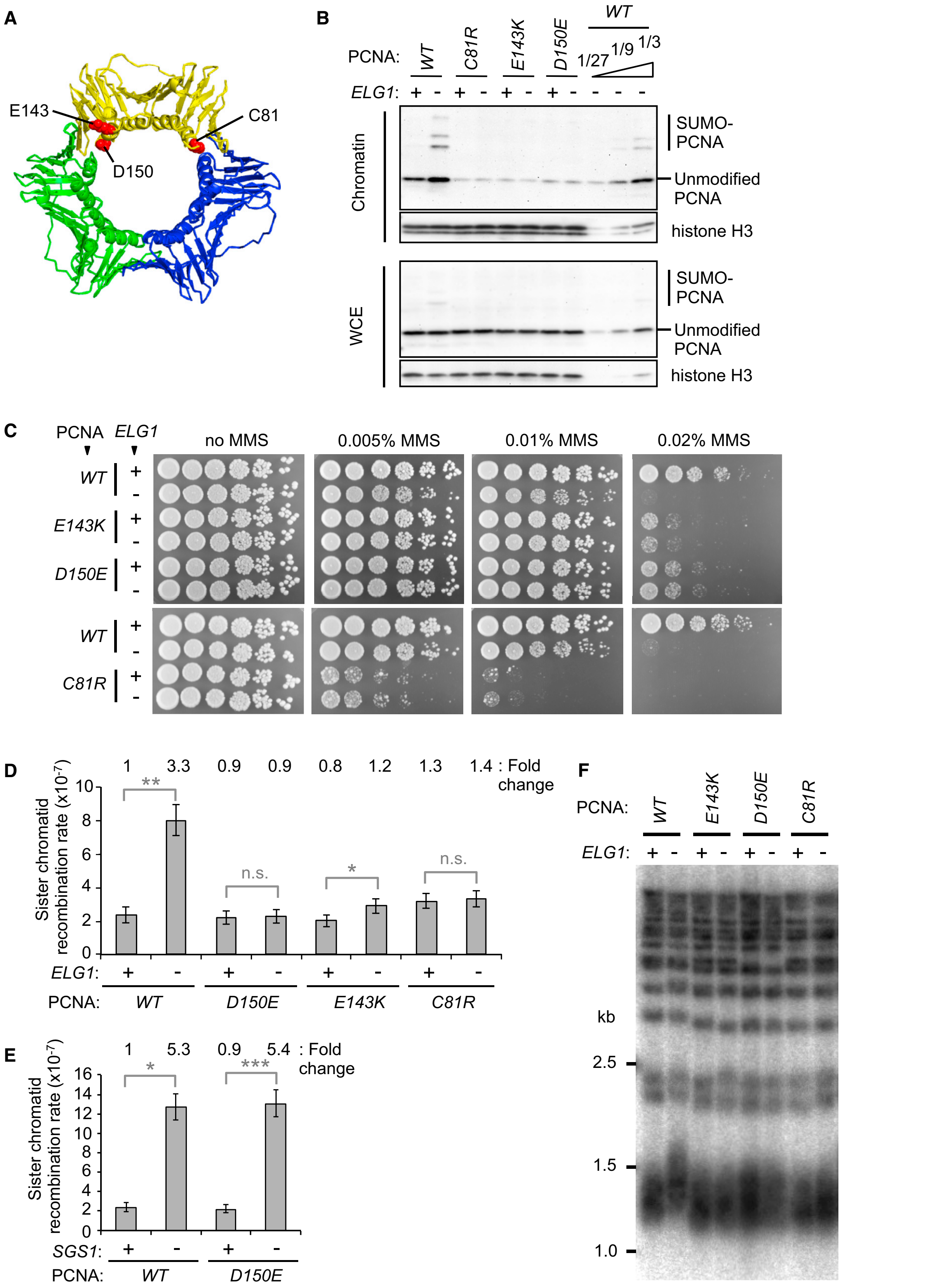

(legend on next page)

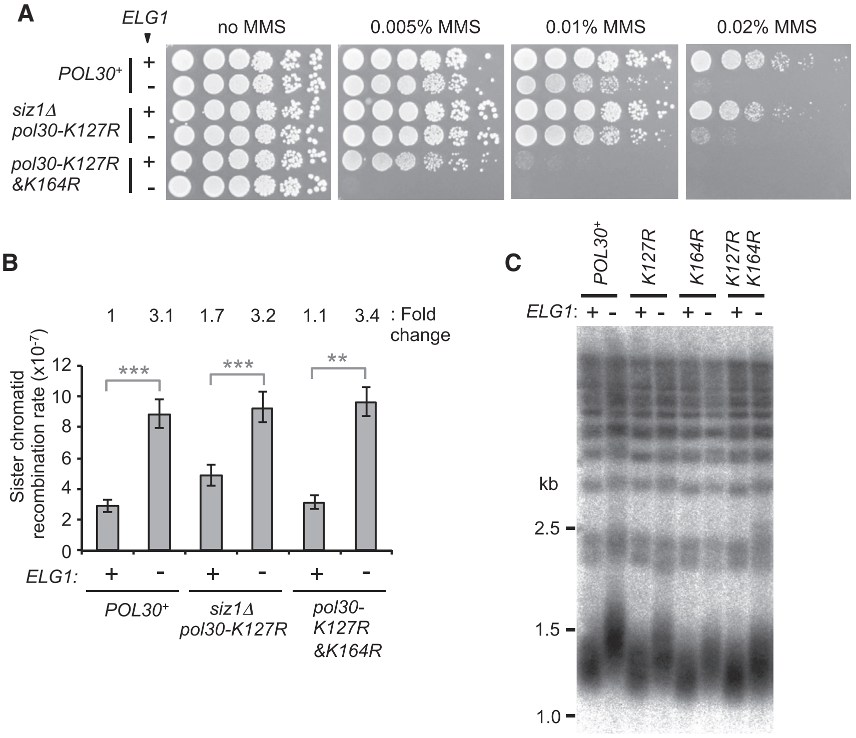

Figure 4. PCNA Modifications Partly Contribute to Abnormal Telomere Elongation but Not the MMS Sensitivity or Increased Sister Chromatid Recombination in *elg1Δ*

(A) Sensitivity of *elg1Δ* to MMS was not rescued by loss of SUMOylation of PCNA (*siz1Δ pol30-K127R*) or loss of both SUMOylation and ubiquitination of PCNA (*pol30-K127&K164R*). 5-fold serial dilutions of the indicated strains were spotted on YPD medium in the presence or absence of MMS. Plates were incubated for 2–3 days at 30°C.

(B) Increased sister chromatid recombination of *elg1Δ* was rescued neither by loss of SUMOylation of PCNA (*siz1Δ pol30-K127R*) nor by loss of SUMOylation and ubiquitination of PCNA (*pol30-K127&K164R*). Fold increase over wild-type is shown. Error bars, 95% confidence intervals. Mann-Whitney; ***$p < 0.0001$; **$p < 0.001$.

(C) Abnormal telomere elongation of *elg1Δ* was partly rescued by mutating modification sites of PCNA (K127R, K164R, or K127R&K164R). Terminal chromosome fragments were detected by probing a Southern blot of XhoI-digested genomic DNA for TG_{1-3} sequence.

G1 phase, to reset PCNA levels before the next round of DNA replication. In *G2/M-ELG1* cells, SUMO-PCNA is over-accumulated in S phase (almost to the same extent as in *elg1Δ*), but, once Elg1 is expressed in G2 phase, SUMO-PCNA disappears (Figures 5Biv and 5C), consistent with unloading of PCNA by G2/M-tagged Elg1. To summarize, we successfully established cell-cycle-regulated alleles of *ELG1* that restrict PCNA removal to M/G1, S, or G2/M phase.

Expression of Elg1 in G2/M Phase Can Rescue the MMS Sensitivity Observed in *elg1Δ*

We used our cell-cycle-regulated *ELG1* alleles to examine whether PCNA retention on DNA at a specific cell-cycle stage causes the genome instability of the *elg1Δ* mutant. Since we already showed that PCNA accumulation on DNA causes MMS sensitivity in *elg1Δ*, we examined the MMS sensitivity of *M/G1-*, *S-*, and *G2/M-ELG1* cells. *M/G1-ELG1* cells were sensitive to MMS, almost to the same extent as the *elg1Δ* mutant (Figure 5D), indicating that Elg1 expression in late M and G1 phases is insufficient to rescue the MMS sensitivity. *M/G1-ELG1* cells show a slow cell-cycle progression in the presence of 0.015% MMS, but M/G1-Elg1 still has the chance to unload PCNA in late M and G1 phases in the presence of 0.015% MMS (Figure S3D). These results suggest that removal of PCNA only in late M and G1 phases is insufficient to rescue the MMS sensitivity. In contrast, *G2/M-ELG1* cells were more resistant to MMS than *M/G1-ELG1* and *elg1Δ* (Figure 5D), indicating that removal of PCNA in G2/M phase (Figures 5Biv and 5C) can largely prevent the sensitivity to MMS observed in *elg1Δ*. These results

Figure 3. Disassembly-Prone Alleles of PCNA Rescue the Increased MMS Sensitivity, Sister Chromatid Recombination, and Telomere Length of *elg1Δ*

(A) Structure of the PCNA trimer (McNally et al., 2010). The positions mutated are highlighted in one of the three subunits (red: residues important for the trimer formation).

(B) The trimer interface PCNA mutants do not accumulate on chromatin in *elg1Δ*. Whole-cell extracts (WCE) and chromatin-enriched fractions (Chromatin) were prepared from cells expressing disassembly-prone PCNA mutants. The disassembly-prone mutants are the only copy of PCNA in these cells. PCNA and histone H3 (loading control) were detected by western blotting. 3-fold dilutions of the WT PCNA *elg1Δ* sample (1/3, 1/9, and 1/27) shown for comparison.

(C) Deletion of *ELG1* does not sensitize cells to MMS in the disassembly-prone PCNA mutant background. 5-fold serial dilutions of *ELG1*+ or *elg1Δ* cells expressing wild-type PCNA or disassembly-prone PCNA mutants (E143K, D150E or C81R) were spotted on YPD medium without or with MMS as indicated. Plates were incubated for 2–3 days at 30°C.

(D) Sister chromatid recombination rate of *ELG1*+ and *elg1Δ* in the wild-type and disassembly-prone PCNA mutant backgrounds was calculated as described in Experimental Procedures. Fold increase over wild-type is shown. Error bars, 95% confidence intervals. Mann-Whitney; **$p < 0.001$; *$p < 0.05$; n.s., $p > 0.05$.

(E) Sister chromatid recombination rate of *SGS1*+ and *sgs1Δ* in the wild-type and disassembly-prone PCNA mutant *D150E*. Fold increase over wild-type is shown. Error bars, 95% confidence intervals. Mann-Whitney; ***$p < 0.0001$; *$p < 0.05$.

(F) Telomere length analysis in wild-type and disassembly-prone PCNA mutant strains (*E143K*, *D150E*, or *C81R*) in the presence or absence of *ELG1*. Terminal chromosome fragments were detected by probing a Southern blot of XhoI-digested genomic DNA for TG_{1-3} sequence. Smeared band at the bottom represents the length of telomeres containing Y' sequence.

See also Figure S2.

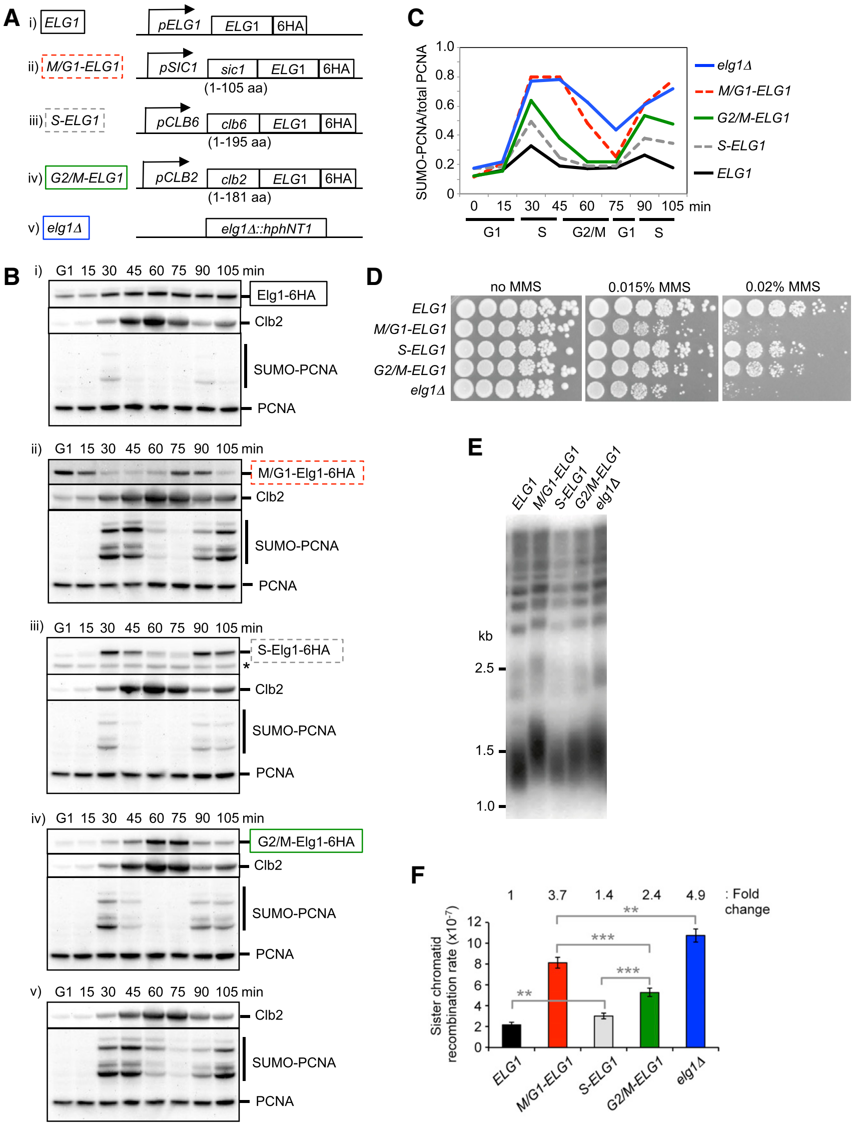

Figure 5. PCNA Retention on DNA into G2/M Phase Causes Sensitivity to MMS, Elongation of Telomeres, and Increased Sister Chromatid Recombination

(A) Schematic of cell-cycle-regulated alleles of *ELG1* that enable restriction of Elg1 to M/G1 (ii), S (iii), or G2/M (iv) phase. Control with wild-type *ELG1* (i) and *elg1*Δ (v) shown for comparison.

(B) Cell-cycle-regulated Elg1 expression and PCNA removal. Cells were arrested in G1 phase for 4.5 hr in alpha-factor and released into the cell cycle by adding pronase. Cells were collected at the indicated time points, and whole-cell extracts were prepared. Elg1 (i) and M/G1- (ii), S- (iii), or G2/M-tagged (iv) Elg1 protein in whole-cell extract was detected by western blot using anti-HA antibody. PCNA and the mitotic cyclin Clb2 was detected with anti-PCNA and anti-Clb2 antibodies, respectively. Asterisk in (iii) indicates degradation products of S-tagged Elg1. All strains are W303 *RAD5*$^{+}$.

(C) Quantification of levels of "chromatin-bound" PCNA. Levels of chromatin-bound PCNA were estimated from levels of SUMOylated PCNA over total PCNA shown in (B). Cell-cycle stages assigned based on times of bud emergence (Figure S3A), PCNA SUMOylation, and Clb2 expression and destruction.

(D) Sensitivity to MMS of the cell-cycle-regulated alleles of *ELG1*. Plates were incubated for 3 days at 30°C. W303 *RAD5*$^{+}$ strains.

(E) Telomere length of the cell-cycle-regulated alleles of *ELG1*. Terminal chromosome fragments were detected by probing a Southern blot of XhoI-digested genomic DNA for TG_{1-3} sequence. S288c strains are shown.

(F) Sister chromatid recombination rates of the cell-cycle-regulated alleles of *ELG1*. Fold changes, compared to *ELG1*$^{+}$ are shown above. Error bars, 95% confidence intervals. Mann-Whitney; ***$p < 0.0001$; **$p < 0.001$. S288c strains carrying the sister chromatid recombination tester locus were used. See also Figure S3.

suggest that PCNA retention on DNA through G2/M phase causes MMS sensitivity. PCNA retention on DNA during S phase causes a mild increase of MMS sensitivity as *G2/M-ELG1* cells are slightly more sensitive than *S-ELG1* cells, and somewhat more sensitive than wild-type *ELG1* cells (Figure 5D). *S-ELG1* cells exhibited mildly increased sensitivity to MMS compared to wild-type *ELG1* (Figure 5D), probably due to the slight delay in PCNA unloading (Figures 5Biii and 5C) because of mildly impaired activity of the tagged Elg1 protein.

The fact that *G2/M-ELG1* cells are not particularly MMS sensitive (Figure 5D) suggests that PCNA does not have to be removed during replication to avoid severe MMS sensitivity, and that some delay in unloading is compatible with fairly normal resistance to MMS. These results suggest that the MMS sensitivity observed in *elg1*Δ is primarily the consequence of retention of PCNA on DNA as cells pass through G2/M phase.

Expression of Elg1 in G2/M Phase Can Partly Rescue Abnormal Elongation of Telomeres Observed in *elg1*Δ

Next, we addressed at which cell-cycle stages expression of Elg1 can prevent abnormal telomere elongation. Telomere elongation occurs normally in late S phase, but can still occur later, in G2/M phase (Diede and Gottschling, 1999). The abnormal telomere elongation of *elg1*Δ was fully rescued in *S-ELG1* cells, and partly rescued in *G2/M-ELG1* cells (Figure 5E). In *M/G1-ELG1* cells, however, telomere length was close to that in *elg1*Δ (Figure 5E), indicating that PCNA removal only at this stage is largely insufficient to prevent inappropriate extension by telomerase. Similar tendencies were observed in a different strain background (Figure S3E). These results suggest that PCNA retention on DNA during S phase leads to some inappropriate elongation of telomeres, while retention through G2/M phase further stimulates the elongation. This is consistent with the idea that PCNA left on

telomeres after replication interferes with the pathways monitoring telomere length.

The Majority of Increased Sister Chromatid Recombination Observed in *elg1*Δ Is Rescued by Expression of Elg1 in G2/M Phase

We then addressed at which cell-cycle-stage PCNA accumulation causes increased sister chromatid recombination. Increased sister chromatid recombination observed in *elg1*Δ was largely rescued in *S-ELG1* cells and moderately rescued in *G2/M-ELG1* cells (Figure 5F). M/G1 phase expression of *ELG1* also caused some rescue of the elevated sister chromatid recombination rate of *elg1*Δ (Figure 5F), contrasting with the inability of *M/G1-ELG1* to rescue MMS sensitivity and telomere length. Abnormal sister chromatid recombination is therefore somewhat stimulated by PCNA accumulation on DNA during S phase, and progressively further stimulated by PCNA retention as cells pass through G2/M phase and into G1 phase. The fact that *M/G1-ELG1* cells exhibit only a mild rescue of increased sister chromatid recombination suggests that the recombination is stimulated by PCNA retention mainly before or during M phase when cells still have paired sister chromatids, but also can be slightly stimulated by PCNA retention in late M or G1 phase (see Discussion). Generally, our results imply that the consequences of PCNA retention on DNA emerge primarily in a postreplicative manner.

Overall, analysis of cell-cycle-regulated alleles reveals that PCNA retention on DNA beyond DNA replication and into G2/M phase is the major cause of genome instability of cells lacking Elg1. While hyper-accumulation of PCNA on DNA during S phase makes some contribution to the genome instability phenotypes of the *elg1*Δ mutant, the largest contribution to genome instability results from continued association of PCNA with DNA into and through G2/M phase.

DISCUSSION

Cells lacking Elg1/ATAD5 exhibit genome instability that leads to cancer (Bell et al., 2011). We have addressed why loss of yeast Elg1 causes genome instability and investigated whether the chromosome maintenance defects result from failure to unload PCNA. Overexpressing PCNA in cells lead to accumulation of PCNA on DNA, which caused sensitivity to MMS and exacerbated increased sister chromatid recombination rates of *elg1*Δ. In contrast, using disassembly-prone PCNA mutants to relieve PCNA accumulation on DNA rescued all the phenotypes of *elg1*Δ tested. These results indicate that PCNA accumulation on DNA is the major cause of genome instability in cells lacking *ELG1*. Using a strategy to restrict Elg1 activity to specific cell-cycle stages, we revealed that prolonged retention of PCNA on DNA following S phase and through G2/M phase is the major cause of the genome instability observed in *elg1*Δ. PCNA unloading by Elg1-RLC is therefore critical for genome maintenance.

Our experiments using *ELG1* truncations revealed that MMS sensitivity generally mirrors failure of PCNA unloading (Figure 1), consistent with our conclusion that PCNA retention on DNA causes MMS sensitivity in *elg1*Δ. Elg1 216–519, however, exhibits a slightly increased resistance to MMS (compared to *elg1*Δ carrying empty vector) despite its poor PCNA unloading activity (Figure 1). This result suggests the possibility that Elg1 plays a minor role in MMS resistance independently of PCNA unloading and the formation of the RFC-like complex, potentially through binding to other proteins such as the Mhf1/Mhf2 histone-like complex that has been reported to bind to the central domain of Elg1 (235–514 aa) (Singh et al., 2013). Increased sensitivity of Elg1 520–791 to MMS (compared to *elg1*Δ carrying empty vector) might be due to competition for small subunits Rfc2-5 with other large subunits Ctf18 and Rad24, the lack of either of which causes increased sensitivity to MMS (Bellaoui et al., 2003). We also found that the N-terminal domain containing the SIMs and PCNA interacting peptide (PIP)-like motif (Parnas et al., 2010) contributes to, but is not essential for PCNA unloading, and is dispensable for resistance to MMS (Figure 1). We observed that the central domain of Elg1 can interact with PCNA (Figure S4), resembling the mode of interaction between RFC and PCNA (Bowman et al., 2004). We suspect that Elg1-RLC binds PCNA in a similar manner to RFC, and the N-terminal domain of Elg1 reinforces the interaction.

To remove PCNA from DNA in *elg1*Δ, we utilized disassembly-prone PCNA mutants that cannot form stable trimers in vitro (Goellner et al., 2014). Despite the trimer instability, cells expressing the disassembly-prone mutants grow normally on YPD without MMS (Figure 3C). It seems likely that these mutants can form trimers in cells sufficiently well that even if they fall off DNA, they can be re-loaded at the exposed 3′ end of DNA repeatedly during DNA synthesis, until the DNA ends are ligated and there is no longer any loading site available. In an *elg1*Δ mutant, PCNA is abnormally retained on DNA even after ligation of Okazaki fragments has occurred (Kubota et al., 2013b, 2015). These trimer instability mutants are therefore ideal for stimulating removal of PCNA in the *elg1*Δ context, where with no persisting Okazaki fragment 3′ end they will no longer be re-loaded. For removing PCNA from DNA in *elg1*Δ without affecting other PCNA functions, the D150E mutant proved the most useful of those we tested as it shows only modest sensitivity to MMS and no increase in sister chromatid recombination rate (Figure 3). In contrast, mutants *C81R*, *V180D*, and *S115P* (known as *pol30-52*) showed increased sensitivity to MMS (Figures 3 and S2C), and the S115P mutation itself caused increased sister chromatid recombination (Figures S2D and S2E). The functionality of the PCNA mutants may depend on how severely their trimer formation is affected in cells and whether the interaction with PCNA-binding proteins is compromised.

Why does PCNA retention on DNA cause an increase in MMS sensitivity and spontaneous sister chromatid recombination? In cells lacking *ELG1*, PCNA retention does not seem to impair Okazaki fragment processing and DNA replication per se (Kubota et al., 2013b; Lee et al., 2013). Instead, we find here that PCNA retention causes genome instability in G2/M phase of the cell cycle (Figure 5), suggesting that the consequences of PCNA retention on DNA emerge in a postreplicative manner, possibly through the abnormal recruitment of interacting partners that initiate inappropriate repair or recombination-mediated events. Alternatively, prolonged presence of PCNA itself or its interacting partners may affect processing of recombination

intermediates, resulting in elevated sister chromatid exchange. Persistent recombination intermediates could also cause chromosome instability in mitosis (Mankouri et al., 2013). However, if removal of PCNA from DNA takes place in G2/M phase by *G2/M-ELG1*, then these recombination intermediates might then still be processed properly by proteins such as Sgs1 and Smc5/6 that can act in G2/M phase (Karras and Jentsch, 2010; Menolfi et al., 2015), so that *G2/M-ELG1* can fairly effectively rescue genome instability of *elg1*Δ.

The increase of sister chromatid recombination that results from PCNA retention in late M and/or G1 phase (Figure 5F) could result from recombination intermediates that persist into late mitosis, causing breakage of catenated DNA and subsequently double-stranded breaks during the next round of DNA synthesis, leading in turn to sister chromatid recombination. Alternatively, PCNA still left on DNA late in the subsequent G1 phase may interfere with the ensuing round of DNA replication.

Abnormal elongation of telomeres in cells lacking *ELG1* is also caused by PCNA retention on DNA (Figure 3F). Telomere elongation is known to occur in late S and G2/M phase (Diede and Gottschling, 1999). We found that removing PCNA from DNA in G2/M phase using the *G2/M-ELG1* allele partly rescued the telomere length defect of *elg1*Δ (Figure 5E). This result presumably reflects that expressing Elg1 during G2/M phase prevents abnormal extension of telomeres late in the cell cycle, but is insufficient to prevent elongation that has already occurred in S phase. We suspect that PCNA retention at the chromosome ends causes a delay in re-formation of specialized telomeric heterochromatin important for inhibiting inappropriate telomere elongation. Post-translationally modified PCNA may further hinder the re-formation of telomeric chromatin, which could explain the partial rescue of abnormal telomere elongation of *elg1*Δ in the unmodifiable PCNA allele. Alternatively, PCNA itself or PCNA-interacting proteins might stimulate telomere elongation through an unknown mechanism. Further work is required to understand how PCNA retention on DNA causes chromosome instability genome-wide and at telomeres.

It is clear that PCNA association with DNA needs to occur in the right place at the right time to coordinate the action of many replisome-associated proteins and repair proteins. Our results using sophisticated genetics in the model organism *S. cerevisiae* reveal that removal of PCNA from DNA by the Elg1-RLC complex after DNA replication (and possibly during and after repair) is critical for genome maintenance. Given the conserved role of the mammalian Elg1 ortholog ATAD5 in PCNA unloading, it is likely the genome instability and carcinogenesis associated with loss of ATAD5 is also caused by PCNA retention on DNA.

EXPERIMENTAL PROCEDURES

Plasmid Construction

The plasmids used are listed in Table S1. The plasmids were constructed using In-fusion Cloning kit (Takara Clontech); see the Supplemental Experimental Procedures for details of specific plasmid constructions. To construct the plasmid pTK31 for M/G1 tagging, the PCR fragment containing the Sic1 promoter (688 bp upstream of ATG) and the coding region of the N terminus of Sic1 (1–105 amino acids) was fused to the plasmid backbone of pRDK1597 (Hombauer et al., 2011) (excluding S-tag) using the In-fusion Cloning kit.

Yeast Strains

S. cerevisiae strains used are listed in Table S2. Epitope tagging and gene disruption were carried out using standard PCR-based gene-insertion methods (Longtine et al., 1998); see the Supplemental Experimental Procedures for details of specific strain constructions. Yeast strains expressing PCNA mutants were gifted from the Kolodner lab (Goellner et al., 2014) or constructed by replacing wild-type PCNA with mutant PCNA (see the Supplemental Experimental Procedures). The plasmids pRS303-*GALp* or pRS303-*GALp-POL30-ADH1t* was integrated at the *LEU2* locus in a derivative of DD452 (Kanellis et al., 2003). S-, G2/M-, or M/G1-tag amplified from the plasmids pRDK1597, pRDK1598 (Hombauer et al., 2011), or pTK31 was integrated to the *ELG1* locus.

Preparation of Whole-Cell Extracts and Chromatin-Enriched Fractions and Western Blotting

Whole-cell extracts and chromatin-enriched fractions were prepared as described previously (Kubota et al., 2015). Western blotting and quantification were performed as described previously (Kubota et al., 2011). Antibodies used were mouse monoclonal anti-PCNA (ab70472, Abcam), rabbit polyclonal anti-histone H3 (ab46765, Abcam), rabbit polyclonal anti-myc (ab9106, Abcam), mouse monoclonal anti-HA (HA.11 clone 16B12, Covance), and rabbit polyclonal anti-Clb2 (y-180, sc-9071, Santa Cruz Biotechnology) antibodies.

Fluctuation Analysis for Sister Chromatid Recombination Rate

The rate (events per cell division) of spontaneous sister chromatid recombination in the indicated strains was determined by fluctuation analysis (Fasullo and Davis, 1987; Liefshitz et al., 1995) using the Ma-Sandri-Sarkar (MSS) maximum-likelihood method (Rosche and Foster, 2000; Sarkar et al., 1992). Each sister chromatid recombination rate was determined using at least 11 independent cultures. Similarly sized colonies grown 2 days at 30°C on YPD (Figures 3D, 3E, 4B, and 5F), YEP-gal (Figure 2C), or SD-Ura (Figures S2D and S2E) plates were transferred to 1–2 ml of liquid YPD, YEP-gal, or SD-Ura and further incubated overnight. After appropriate dilution the cells were plated on YPD to count viable cells, and SD-His or SD-His-Ura to measure sister chromatid recombination. Colonies were counted after 3 days. The 95% confidence intervals were calculated as described previously (Rosche and Foster, 2000). Mann-Whitney tests were performed to report the two-tailed p values (http://vassarstats.net/utest.html).

Telomere Length Analysis

Genomic DNA was digested with XhoI, separated on a 1% agarose gel, and transferred to neutral membrane (MP Biomedicals) by Southern blotting. Terminal restriction fragments were detected using a probe directed against the TG repeats.

SUPPLEMENTAL INFORMATION

Supplemental Information includes Supplemental Experimental Procedures, four figures, and two tables and can be found with this article online at http://dx.doi.org/10.1016/j.celrep.2016.06.030.

AUTHOR CONTRIBUTIONS

C.J. and T.K. performed most experiments. V.K.G. constructed strains and performed immunoprecipitation. C.J. and T.K. conceived and designed the experiments. T.S.T. wrote a program for calculating recombination rates and advised on recombination assay. T.K. wrote the paper.

ACKNOWLEDGMENTS

We thank Richard Kolodner, Grant Brown, and Daniel Durocher for strains and plasmids. We thank Anne Donaldson, Alexander Lorenz, and Shin-ichiro Hiraga from University of Aberdeen for careful reading of the manuscript. Research in T.K.'s lab is supported by Medical Research Council Career Development Fellowship L019698/1. V.K.G. was supported by Biotechnology and Biological Sciences Research Council grant K006304/1. T.S.T. was

supported by grants from the Ministry of Education, Culture, Sports, Science and Technology of Japan (nos. 23131507 and 25131712).

Received: February 5, 2016
Revised: April 28, 2016
Accepted: June 3, 2016
Published: June 30, 2016

REFERENCES

Aguilera, A., and Gómez-González, B. (2008). Genome instability: a mechanistic view of its causes and consequences. Nat. Rev. Genet. *9*, 204–217.

Andersen, P.L., Xu, F., and Xiao, W. (2008). Eukaryotic DNA damage tolerance and translesion synthesis through covalent modifications of PCNA. Cell Res. *18*, 162–173.

Ashton, T.M., and Hickson, I.D. (2010). Yeast as a model system to study RecQ helicase function. DNA Repair (Amst.) *9*, 303–314.

Ayyagari, R., Impellizzeri, K.J., Yoder, B.L., Gary, S.L., and Burgers, P.M. (1995). A mutational analysis of the yeast proliferating cell nuclear antigen indicates distinct roles in DNA replication and DNA repair. Mol. Cell. Biol. *15*, 4420–4429.

Banerjee, S., and Myung, K. (2004). Increased genome instability and telomere length in the elg1-deficient Saccharomyces cerevisiae mutant are regulated by S-phase checkpoints. Eukaryot. Cell *3*, 1557–1566.

Baple, E.L., Chambers, H., Cross, H.E., Fawcett, H., Nakazawa, Y., Chioza, B.A., Harlalka, G.V., Mansour, S., Sreekantan-Nair, A., Patton, M.A., et al. (2014). Hypomorphic PCNA mutation underlies a human DNA repair disorder. J. Clin. Invest. *124*, 3137–3146.

Bell, D.W., Sikdar, N., Lee, K.Y., Price, J.C., Chatterjee, R., Park, H.D., Fox, J., Ishiai, M., Rudd, M.L., Pollock, L.M., et al.; NISC Comparative Sequencing Program (2011). Predisposition to cancer caused by genetic and functional defects of mammalian Atad5. PLoS Genet. *7*, e1002245.

Bellaoui, M., Chang, M., Ou, J., Xu, H., Boone, C., and Brown, G.W. (2003). Elg1 forms an alternative RFC complex important for DNA replication and genome integrity. EMBO J. *22*, 4304–4313.

Ben-Aroya, S., Koren, A., Liefshitz, B., Steinlauf, R., and Kupiec, M. (2003). ELG1, a yeast gene required for genome stability, forms a complex related to replication factor C. Proc. Natl. Acad. Sci. USA *100*, 9906–9911.

Bienko, M., Green, C.M., Crosetto, N., Rudolf, F., Zapart, G., Coull, B., Kannouche, P., Wider, G., Peter, M., Lehmann, A.R., et al. (2005). Ubiquitin-binding domains in Y-family polymerases regulate translesion synthesis. Science *310*, 1821–1824.

Bowman, G.D., O'Donnell, M., and Kuriyan, J. (2004). Structural analysis of a eukaryotic sliding DNA clamp-clamp loader complex. Nature *429*, 724–730.

Davidson, M.B., and Brown, G.W. (2008). The N- and C-termini of Elg1 contribute to the maintenance of genome stability. DNA Repair (Amst.) *7*, 1221–1232.

Davies, A.A., Huttner, D., Daigaku, Y., Chen, S., and Ulrich, H.D. (2008). Activation of ubiquitin-dependent DNA damage bypass is mediated by replication protein a. Mol. Cell *29*, 625–636.

Dieckman, L.M., Boehm, E.M., Hingorani, M.M., and Washington, M.T. (2013). Distinct structural alterations in proliferating cell nuclear antigen block DNA mismatch repair. Biochemistry *52*, 5611–5619.

Diede, S.J., and Gottschling, D.E. (1999). Telomerase-mediated telomere addition in vivo requires DNA primase and DNA polymerases alpha and delta. Cell *99*, 723–733.

Duffy, C.M., Hilbert, B.J., and Kelch, B.A. (2016). A disease-causing variant in PCNA disrupts a promiscuous protein binding site. J. Mol. Biol. *428*, 1023–1040.

Fasullo, M.T., and Davis, R.W. (1987). Recombinational substrates designed to study recombination between unique and repetitive sequences in vivo. Proc. Natl. Acad. Sci. USA *84*, 6215–6219.

Goellner, E.M., Smith, C.E., Campbell, C.S., Hombauer, H., Desai, A., Putnam, C.D., and Kolodner, R.D. (2014). PCNA and Msh2-Msh6 activate an Mlh1-Pms1 endonuclease pathway required for Exo1-independent mismatch repair. Mol. Cell *55*, 291–304.

Gomes, X.V., and Burgers, P.M. (2001). ATP utilization by yeast replication factor C. I. ATP-mediated interaction with DNA and with proliferating cell nuclear antigen. J. Biol. Chem. *276*, 34768–34775.

Hoege, C., Pfander, B., Moldovan, G.L., Pyrowolakis, G., and Jentsch, S. (2002). RAD6-dependent DNA repair is linked to modification of PCNA by ubiquitin and SUMO. Nature *419*, 135–141.

Hombauer, H., Srivatsan, A., Putnam, C.D., and Kolodner, R.D. (2011). Mismatch repair, but not heteroduplex rejection, is temporally coupled to DNA replication. Science *334*, 1713–1716.

Kanellis, P., Agyei, R., and Durocher, D. (2003). Elg1 forms an alternative PCNA-interacting RFC complex required to maintain genome stability. Curr. Biol. *13*, 1583–1595.

Karras, G.I., and Jentsch, S. (2010). The RAD6 DNA damage tolerance pathway operates uncoupled from the replication fork and is functional beyond S phase. Cell *141*, 255–267.

Kelch, B.A., Makino, D.L., O'Donnell, M., and Kuriyan, J. (2011). How a DNA polymerase clamp loader opens a sliding clamp. Science *334*, 1675–1680.

Kubota, T., Hiraga, S., Yamada, K., Lamond, A.I., and Donaldson, A.D. (2011). Quantitative proteomic analysis of chromatin reveals that Ctf18 acts in the DNA replication checkpoint. Mol. Cell. Proteomics *10*, 005561.

Kubota, T., Myung, K., and Donaldson, A.D. (2013a). Is PCNA unloading the central function of the Elg1/ATAD5 replication factor C-like complex? Cell Cycle *12*, 2570–2579.

Kubota, T., Nishimura, K., Kanemaki, M.T., and Donaldson, A.D. (2013b). The Elg1 replication factor C-like complex functions in PCNA unloading during DNA replication. Mol. Cell *50*, 273–280.

Kubota, T., Katou, Y., Nakato, R., Shirahige, K., and Donaldson, A.D. (2015). Replication-Coupled PCNA Unloading by the Elg1 Complex Occurs Genome-wide and Requires Okazaki Fragment Ligation. Cell Rep. *12*, 774–787.

Kuchenbaecker, K.B., Ramus, S.J., Tyrer, J., Lee, A., Shen, H.C., Beesley, J., Lawrenson, K., McGuffog, L., Healey, S., Lee, J.M., et al.; EMBRACE; GEMO Study Collaborators; Breast Cancer Family Registry; HEBON; KConFab Investigators; Australian Cancer Study (Ovarian Cancer Investigators); Australian Ovarian Cancer Study Group; Consortium of Investigators of Modifiers of BRCA1 and BRCA2 (2015). Identification of six new susceptibility loci for invasive epithelial ovarian cancer. Nat. Genet. *47*, 164–171.

Lau, P.J., Flores-Rozas, H., and Kolodner, R.D. (2002). Isolation and characterization of new proliferating cell nuclear antigen (POL30) mutator mutants that are defective in DNA mismatch repair. Mol. Cell. Biol. *22*, 6669–6680.

Lee, K.Y., Fu, H., Aladjem, M.I., and Myung, K. (2013). ATAD5 regulates the lifespan of DNA replication factories by modulating PCNA level on the chromatin. J. Cell Biol. *200*, 31–44.

Liefshitz, B., Parket, A., Maya, R., and Kupiec, M. (1995). The role of DNA repair genes in recombination between repeated sequences in yeast. Genetics *140*, 1199–1211.

Longtine, M.S., McKenzie, A., 3rd, Demarini, D.J., Shah, N.G., Wach, A., Brachat, A., Philippsen, P., and Pringle, J.R. (1998). Additional modules for versatile and economical PCR-based gene deletion and modification in Saccharomyces cerevisiae. Yeast *14*, 953–961.

Mailand, N., Gibbs-Seymour, I., and Bekker-Jensen, S. (2013). Regulation of PCNA-protein interactions for genome stability. Nat. Rev. Mol. Cell Biol. *14*, 269–282.

Mankouri, H.W., Huttner, D., and Hickson, I.D. (2013). How unfinished business from S-phase affects mitosis and beyond. EMBO J. *32*, 2661–2671.

Maradeo, M.E., and Skibbens, R.V. (2009). The Elg1-RFC clamp-loading complex performs a role in sister chromatid cohesion. PLoS ONE *4*, e4707.

McNally, R., Bowman, G.D., Goedken, E.R., O'Donnell, M., and Kuriyan, J. (2010). Analysis of the role of PCNA-DNA contacts during clamp loading. BMC Struct. Biol. *10*, 3.

Menolfi, D., Delamarre, A., Lengronne, A., Pasero, P., and Branzei, D. (2015). Essential Roles of the Smc5/6 Complex in Replication through Natural Pausing Sites and Endogenous DNA Damage Tolerance. Mol. Cell *60*, 835–846.

Moldovan, G.L., Pfander, B., and Jentsch, S. (2007). PCNA, the maestro of the replication fork. Cell *129*, 665–679.

Negrini, S., Gorgoulis, V.G., and Halazonetis, T.D. (2010). Genomic instability–an evolving hallmark of cancer. Nat. Rev. Mol. Cell Biol. *11*, 220–228.

Papouli, E., Chen, S., Davies, A.A., Huttner, D., Krejci, L., Sung, P., and Ulrich, H.D. (2005). Crosstalk between SUMO and ubiquitin on PCNA is mediated by recruitment of the helicase Srs2p. Mol. Cell *19*, 123–133.

Parker, J.L., and Ulrich, H.D. (2009). Mechanistic analysis of PCNA poly-ubiquitylation by the ubiquitin protein ligases Rad18 and Rad5. EMBO J. *28*, 3657–3666.

Parker, J.L., Bucceri, A., Davies, A.A., Heidrich, K., Windecker, H., and Ulrich, H.D. (2008). SUMO modification of PCNA is controlled by DNA. EMBO J. *27*, 2422–2431.

Parnas, O., Zipin-Roitman, A., Mazor, Y., Liefshitz, B., Ben-Aroya, S., and Kupiec, M. (2009). The ELG1 clamp loader plays a role in sister chromatid cohesion. PLoS ONE *4*, e5497.

Parnas, O., Zipin-Roitman, A., Pfander, B., Liefshitz, B., Mazor, Y., Ben-Aroya, S., Jentsch, S., and Kupiec, M. (2010). Elg1, an alternative subunit of the RFC clamp loader, preferentially interacts with SUMOylated PCNA. EMBO J. *29*, 2611–2622.

Parnas, O., Amishay, R., Liefshitz, B., Zipin-Roitman, A., and Kupiec, M. (2011). Elg1, the major subunit of an alternative RFC complex, interacts with SUMO-processing proteins. Cell Cycle *10*, 2894–2903.

Pfander, B., Moldovan, G.L., Sacher, M., Hoege, C., and Jentsch, S. (2005). SUMO-modified PCNA recruits Srs2 to prevent recombination during S phase. Nature *436*, 428–433.

Rosche, W.A., and Foster, P.L. (2000). Determining mutation rates in bacterial populations. Methods *20*, 4–17.

Sarkar, S., Ma, W.T., and Sandri, G.H. (1992). On fluctuation analysis: a new, simple and efficient method for computing the expected number of mutants. Genetica *85*, 173–179.

Sheaff, R.J., and Roberts, J.M. (1996). End of the line: proteolytic degradation of cyclin-dependent kinase inhibitors. Chem. Biol. *3*, 869–873.

Shiomi, Y., and Nishitani, H. (2013). Alternative replication factor C protein, Elg1, maintains chromosome stability by regulating PCNA levels on chromatin. Genes Cells *18*, 946–959.

Singh, S., Shemesh, K., Liefshitz, B., and Kupiec, M. (2013). Genetic and physical interactions between the yeast ELG1 gene and orthologs of the Fanconi anemia pathway. Cell Cycle *12*, 1625–1636.

Smolikov, S., Mazor, Y., and Krauskopf, A. (2004). ELG1, a regulator of genome stability, has a role in telomere length regulation and in silencing. Proc. Natl. Acad. Sci. USA *101*, 1656–1661.

Stelter, P., and Ulrich, H.D. (2003). Control of spontaneous and damage-induced mutagenesis by SUMO and ubiquitin conjugation. Nature *425*, 188–191.

Ulrich, H.D. (2013). New insights into replication clamp unloading. J. Mol. Biol. *425*, 4727–4732.

Urulangodi, M., Sebesta, M., Menolfi, D., Szakal, B., Sollier, J., Sisakova, A., Krejci, L., and Branzei, D. (2015). Local regulation of the Srs2 helicase by the SUMO-like domain protein Esc2 promotes recombination at sites of stalled replication. Genes Dev. *29*, 2067–2080.

Cell Reports, Volume *16*

Supplemental Information

PCNA Retention on DNA into G2/M Phase Causes Genome Instability in Cells Lacking Elg1

Catherine Johnson, Vamsi K. Gali, Tatsuro S. Takahashi, and Takashi Kubota

Supplemental Experimental Procedures

Plasmid construction

The plasmids used are listed in Table S1. The plasmid pTK23 containing *NHP6B*, *TRP1* and *POL30* was constructed by fusing PCR products amplified from genomic DNA and the backbone of pRS304 using the In-fusion Cloning kit (Takara Clontech). Similarly, the plasmid pTK24 containing *NHP6B*, *LEU2*, and *POL30* was constructed using the In-fusion Cloning kit. For constructing the plasmids containing PCNA mutant alleles (pTK26, 27, 28, and 30), *pol30* mutant alleles were amplified from genomic DNA prepared from existing *pol30* mutants (Goellner et al., 2014; Windecker and Ulrich, 2008). To construct the plasmid pTK31 for M/G1-tagging, the PCR fragment containing the Sic1 promoter (688 bp upstream of ATG) and the coding region of the N-terminus of Sic1 (1-105 amino acids) was fused to the plasmid backbone of pRDK1597 (Hombauer et al., 2011) (including natNT2, excluding S-tag) using the In-fusion Cloning kit. The plasmid pRS303-*GALp-POL30-ADH1t* was constructed by fusing PCR fragments of *POL30* and the terminator of *ADH1* to pRS303-*GALp* using the In-fusion Cloning kit.

Yeast strains

S. cerevisiae strains used are listed in Table S2. Epitope tagging and gene disruption were carried out using standard PCR-based gene-insertion methods (Longtine et al., 1998). Yeast strains expressing trimer interface PCNA mutants (Goellner et al., 2014) were gifted from the Kolodner lab, and were used for initial screening for the MMS sensitivity and their retentions on chromatin (Figure 3B and Figure S2C). Since those strains have a disrupted *NHP6B* gene (located upstream of the *POL30* locus) caused

by insertion of the *LEU2* gene, we constructed strains expressing PCNA mutants with functional Nhp6B using the plasmids pTK23, 24, 26, 27, 28, and 30. The *NHP6B* gene disrupted by the *LEU2* gene was replaced with a fragment containing the intact *NHP6B* gene followed by the *TRP1* gene (that was amplified by PCR from the plasmid pTK23). Strains expressing PCNA-K164R, PCNA-K127R, PCNA-K127R&K164R, and PCNA-D150E were constructed by replacing the *POL30* gene with fragments containing the intact *NHP6B* followed by *LEU2*, and the PCNA mutant alleles (that were prepared by digestion of pTK26, pTK27, pTK28 and pTK30 by BanII). The mutations were confirmed by sequencing the amplified fragments from genomic DNA.

For testing the effect of PCNA overexpression on sister chromatid recombination, the plasmids pRS303-*GALp* or pRS303-*GALp-POL30-ADH1t* was integrated at the *LEU2* locus in a derivative of DD452 (Kanellis et al., 2003). To construct the sister chromatid recombination tester strains containing PCNA mutant alleles or cell-cycle-regulated *ELG1* alleles, the sister chromatid recombination tester locus (containing two truncated fragments of *HIS3* and the *URA3* gene) was amplified from genomic DNA prepared from a derivative of DD452 and integrated into the *TRP1* locus of cells containing PCNA mutant alleles or RDKY5964 (a parent strain of PCNA mutants or cell-cycle-regulated *ELG1* cells).

To construct strains expressing S-, G2/M-, or M/G1-tagged Elg1, the plasmids pRDK1597, pRDK1598 (Hombauer et al., 2011), or pTK31 were used as template DNAs in PCR reactions. These PCR products targeted the chromosomal *ELG1* locus, leaving the *ELG1* gene under control of the cyclin or a CDK inhibitor promoter and

fusing the N-terminal domains of these cyclins or CDK inhibitor to the N-terminus of Elg1 at amino acid 2.

Fig. S1

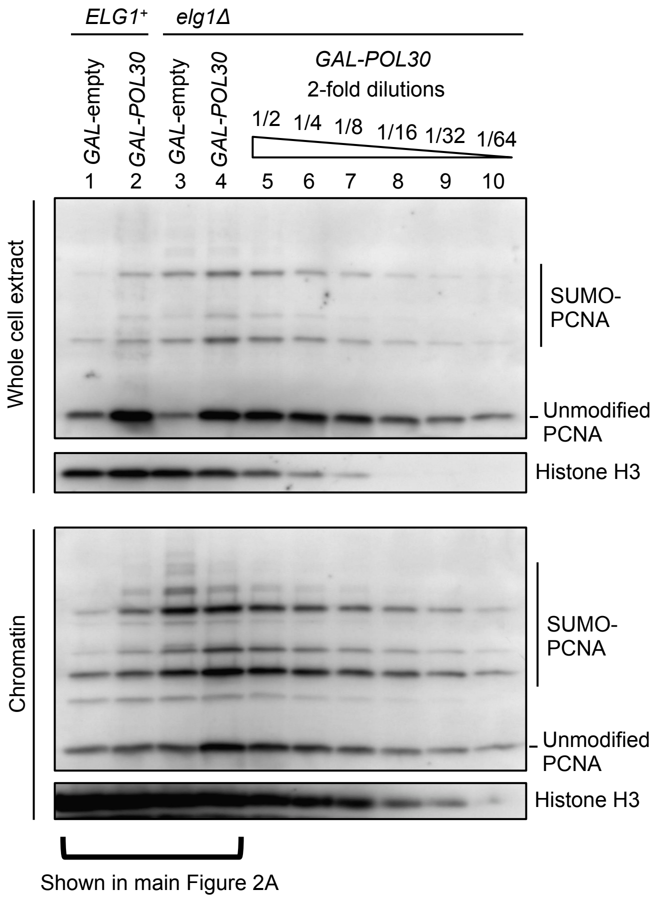
ELG1+
elg1Δ
GAL-empty
GAL-POL30
GAL-empty
GAL-POL30
GAL-POL30
2-fold dilutions
1/2 1/4 1/8 1/16 1/32 1/64
1 2 3 4 5 6 7 8 9 10
Whole cell extract
SUMO-PCNA
Unmodified PCNA
Histone H3
Chromatin
SUMO-PCNA
Unmodified PCNA
Histone H3
Shown in main Figure 2A

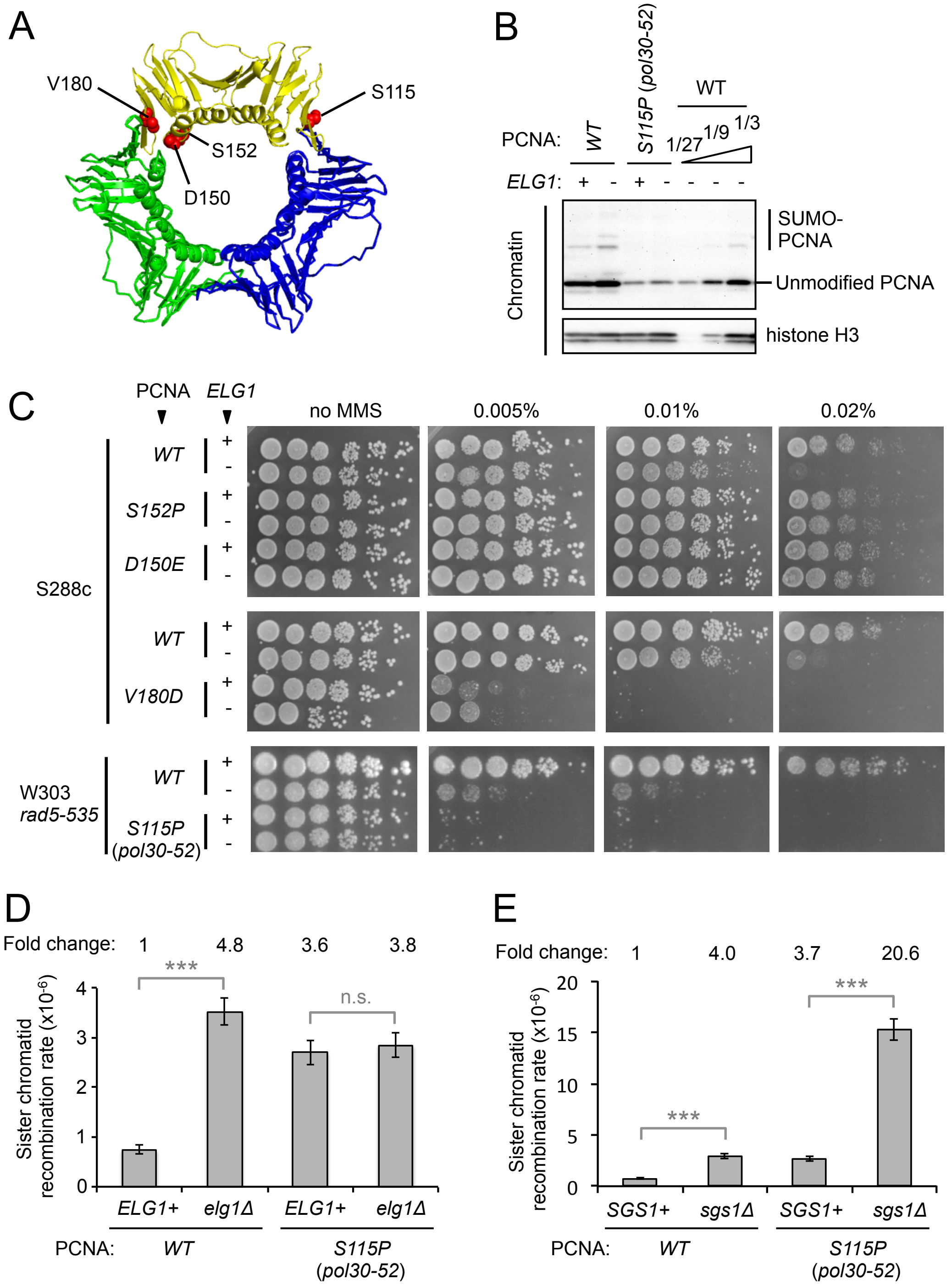
A
V180
S115
S152
D150
B
PCNA:
WT
S115P (pol30-52)
WT
1/27
1/9
1/3
ELG1:
Chromatin
SUMO-PCNA
Unmodified PCNA
histone H3
C
PCNA
ELG1
no MMS
0.005%
0.01%
0.02%
S288c
WT
S152P
D150E
V180D
W303 rad5-535
S115P (pol30-52)
D
Fold change: 1 4.8 3.6 3.8
***
n.s.
Sister chromatid recombination rate (x10-6)
ELG1+
elg1Δ
PCNA: WT
S115P (pol30-52)
E
Fold change: 1 4.0 3.7 20.6
SGS1+
sgs1Δ

Fig. S3

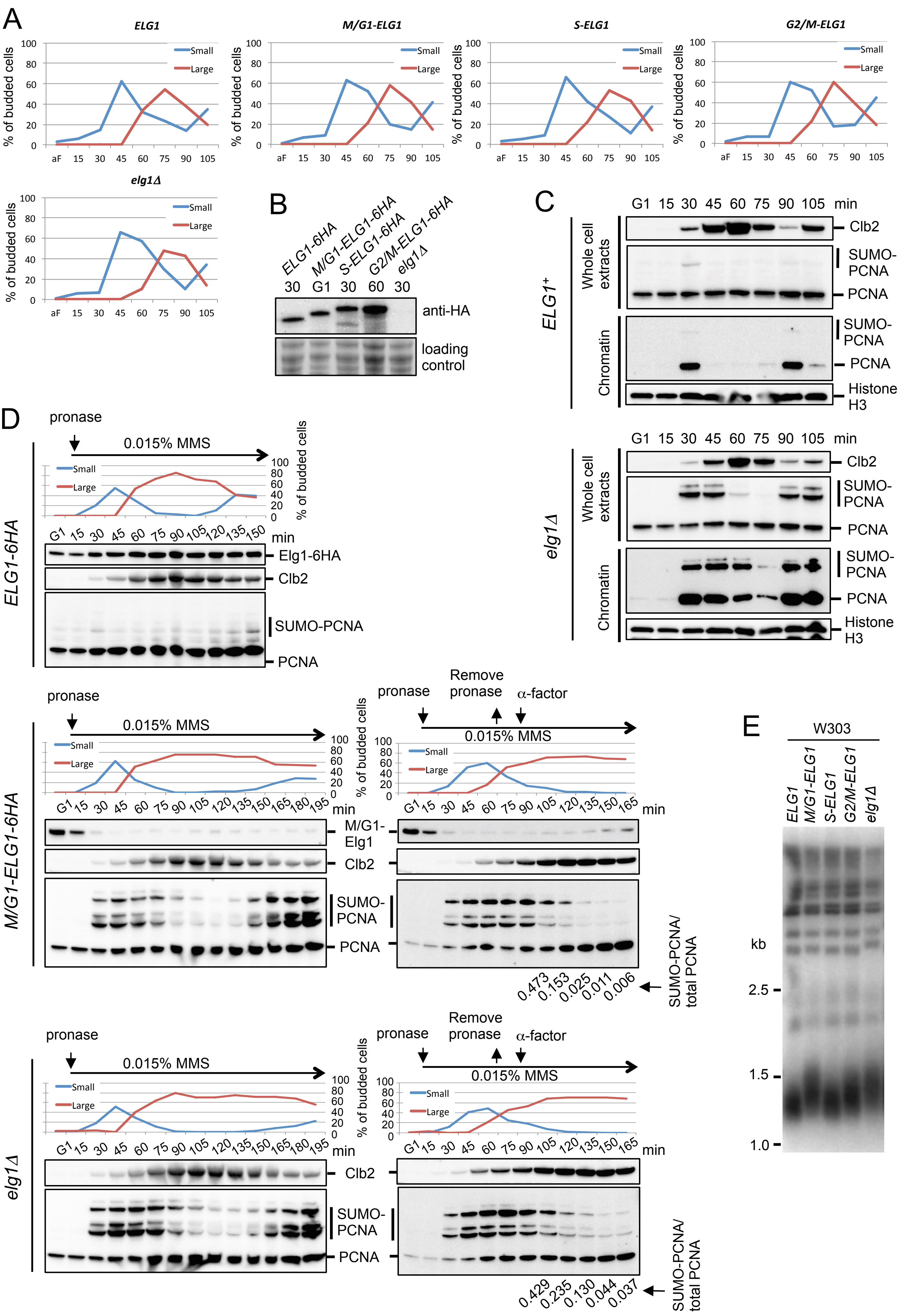

A
ELG1
M/G1-ELG1
S-ELG1
G2/M-ELG1
elg1Δ
% of budded cells
Small
Large
aF 15 30 45 60 75 90 105
B
ELG1-6HA
M/G1-ELG1-6HA
S-ELG1-6HA
G2/M-ELG1-6HA
elg1Δ
30 G1 30 60 30
anti-HA
loading control
C
ELG1+
elg1Δ
Whole cell extracts
Chromatin
G1 15 30 45 60 75 90 105 min
Clb2
SUMO-PCNA
PCNA
Histone H3
D
pronase
0.015% MMS
ELG1-6HA
M/G1-ELG1-6HA
elg1Δ
% of budded cells
Elg1-6HA
M/G1-Elg1
Clb2
SUMO-PCNA
PCNA
min
Remove pronase
α-factor
SUMO-PCNA/total PCNA
0.473 0.153 0.025 0.011 0.006
0.429 0.235 0.130 0.044 0.037
E
W303
ELG1
M/G1-ELG1
S-ELG1
G2/M-ELG1
elg1Δ
kb
2.5
1.5
1.0

Fig. S4

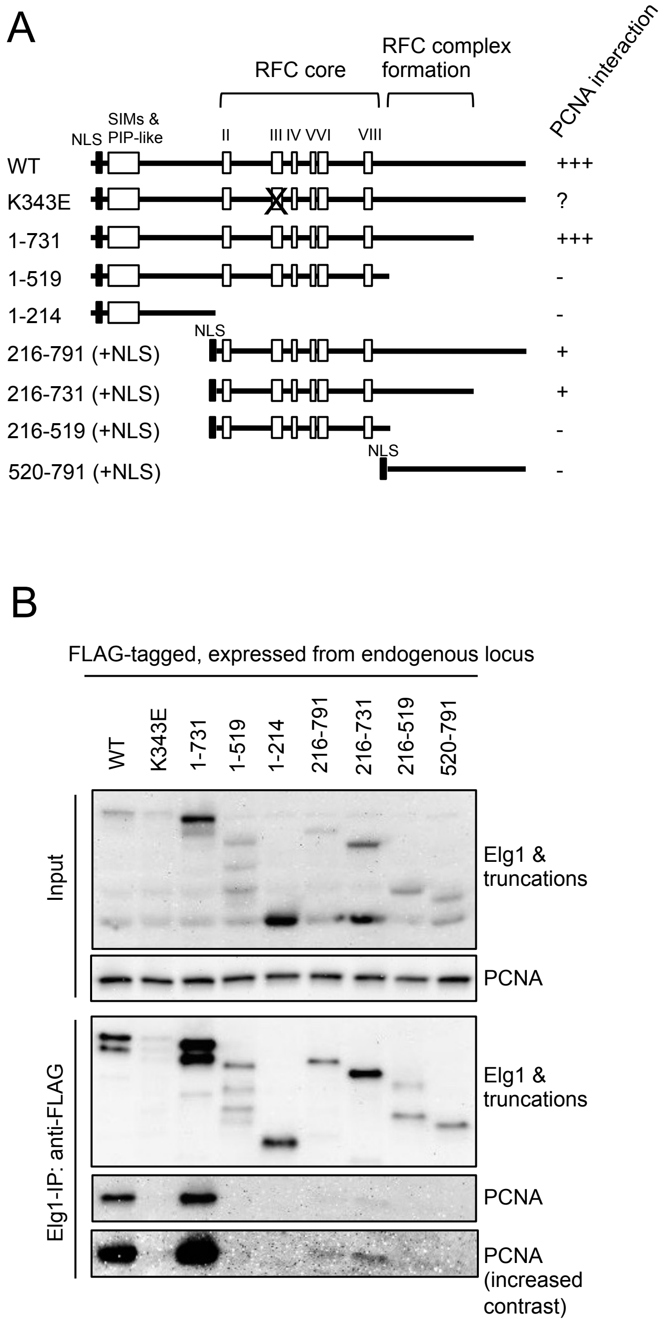

A
RFC core
RFC complex formation
PCNA interaction
NLS
SIMs & PIP-like
II
III IV VVI
VIII
WT
+++
K343E
?
1-731
+++
1-519
-
1-214
-
216-791 (+NLS)
+
216-731 (+NLS)
+
216-519 (+NLS)
-
520-791 (+NLS)
-
B
FLAG-tagged, expressed from endogenous locus
WT
K343E
1-731
1-519
1-214
216-791
216-731
216-519
520-791
Input
Elg1 & truncations
PCNA
Elg1-IP: anti-FLAG
Elg1 & truncations
PCNA
PCNA (increased contrast)

Supplemental Figure legends

Figure S1, related to main Figure 2. Western blots to quantify PCNA amounts. Whole cell extracts and chromatin-enriched fractions were prepared as described in the Figure legend of main Figure 2. Two-fold serial dilutions of protein samples prepared from *GAL-POL30 elg1Δ* (lanes 5-10) were shown here and used for quantification. For the quantification, the intensity of all PCNA bands in each lane was measured and the intensity of the empty lane (not shown) was subtracted from it. Using 2-fold serial dilutions, relative amounts to *ELG1*$^+$ *GAL-empty* were calculated. The values normalised to histone H3 are shown in main Figure 2A.

Figure S2, related to main Figure 3. The trimer interface mutant alleles of PCNA.
(**A**) Structure of the PCNA trimer. The positions mutated are highlighted.
(**B**) The trimer interface PCNA mutant S115P does not accumulate on chromatin in *elg1*Δ. Chromatin-enriched fractions (Chromatin) were prepared from cells expressing wild-type PCNA or the S115P mutant. PCNA and histone H3 (loading control) were detected by western blotting.
(**C**) Deletion of *ELG1* does not sensitise cells to MMS in the trimer interface PCNA mutant background. Five-fold serial dilutions of *ELG1*$^+$ or *elg1*Δ expressing wild-type or trimer interface mutant PCNA (S152P, D150E, V180D or S115P) were spotted on YPD medium plus or minus MMS. Cells were incubated for 2-3 days at 30°C.
(**D**) Sister chromatid recombination rate of *ELG1*$^+$ and *elg1*Δ in the *S115P* background. *S115P* itself showed increased sister chromatid recombination, but deleting *ELG1* did not increase it further. Error bars, 95% confidence intervals. Mann-Whitney; ***, p-value < 0.0001; n.s., p-value > 0.05.
(**E**) In contrast to *elg1Δ*, sister chromatid recombination rate of *sgs1*Δ was further increased by the trimer interface mutant allele (*S115P*) of PCNA. Error bars, 95% confidence intervals. Mann-Whitney; ***, p-value < 0.0001.

Figure S3, related to main Figure 5. Analysis of cell-cycle-regulated *ELG1* strains.
(**A**) Percentage of small- or large-budded cells in cell-cycle-regulated *ELG1* strains shown in the main Figure 5B.
(**B**) Comparison of expression levels of Elg1-6HA (at 30 min after release) and M/G1-(G1-arrested), S- (30 min), and G2/M-tagged (60 min) Elg1-6HA.
(**C**) The levels of chromatin-bound PCNA correlate with those of SUMO-PCNA in whole cell extracts. Wild-type and *elg1Δ* cells were released from G1 (alpha-factor arrest) into the cell cycle. Samples collected at the indicated time points were kept on ice and 0.1% sodium azide was added. All samples were processed together to prepare whole cell extracts and chromatin fractions.
(**D**) Analysis of *M/G1-ELG1* cells in 0.015% MMS. *WT*, *M/G1-ELG1,* and *elg1Δ* cells were released from G1 into the cell cycle in the presence of 0.015% MMS and whole cell extracts were prepared at the indicated time points (left panels). *M/G1-ELG1* and *elg1Δ* show a slow cell cycle progression in 0.015% MMS. It appears that M/G1-Elg1 can unload PCNA in late M and G1 (120 min) although its expression was not observed clearly, probably due to asynchrony. Reappearance of SUMO-PCNA at the 150-195 min samples indicates that some cells enter the second S phase. To see PCNA unloading by M/G1-Elg1 in the late M and the second G1 phases in the presence of 0.015% MMS more clearly, *M/G1-ELG1* and *elg1Δ* cells were released from G1 into the cell cycle in 0.015% MMS and then arrested in the second G1 phase (right panels). To arrest cells in the second G1 phase, pronase was removed

by washing and resuspending cells in YPD containing 0.015% (after taking the 60 min samples), and then an excess amount of alpha-factor was added to the culture (after taking the 75 min samples). When M/G1-Elg1 was expressed in the late M and the second G1 phases, faster reduction of SUMO-PCNA was observed (compared to *elg1Δ*), indicating that M/G1-Elg1 has the chance to unload PCNA in the presence of MMS. Quantification of SUMO-PCNA/total PCNA at the 105-165 min time points is shown below.
(**E**) Telomere length of the cell-cycle-regulated alleles of *ELG1*. W303 *RAD5*$^{+}$ strains are shown.

Figure S4, related to main Figure 1. Elg1 can interact with PCNA through its central domain, and the interaction is reinforced by its N-terminal domain.
(**A**) Schematic structure of Elg1 and its mutants.
(**B**) Immunoprecipitation assay to determine the domain that can interact with PCNA. Cells expressing FLAG-tagged Elg1 or its truncations from endogenous locus were used. Immunoprecipitation was performed as described previously (Kubota et al. 2011; Kubota et al. 2013) with minor modifications. Briefly, approximately 1 x 10^9 cells were spheroplasted and lysed in 600 μl low-salt buffer (which contains 50 mM potassium acetate). Soluble lysates were prepared by centrifugation following Benzonase treatment and then used for immunoprecipitation with anti-FLAG antibody coupled to Dynabeads Protein G. FLAG-tagged proteins and associated proteins were eluted using 3xFLAG peptides.

Supplemental Table S1. Plasmids used in this study, related to Figures 1-5		
**Plasmid name**	**Relevant Genotype**	**Reference**
pRS315	*amp^r CEN6 ARSH4 LEU2*	Sikorski and Hieter 1989
pRS315-*ELG1-13myc*	*amp^r CEN6 ARSH4 LEU2 P_{ELG1}-ELG1-13myc*	Davidson and Brown 2008
pRS315-*elg1(1-731)-13myc*	*amp^r CEN6 ARSH4 LEU2 P_{ELG1}-elg1(1-731)-13myc*	Davidson and Brown 2008
pRS315-*elg1(1-519)-13myc*	*amp^r CEN6 ARSH4 LEU2 P_{ELG1}-elg1(1-519)-13myc*	Davidson and Brown 2008
pRS315-*elg1(1-214)-13myc*	*amp^r CEN6 ARSH4 LEU2 P_{ELG1}-elg1(1-214)-13myc*	Davidson and Brown 2008
pRS315-*elg1(NLS-216-791)-13myc*	*amp^r CEN6 ARSH4 LEU2 P_{ELG1}-elg1(NLS-216-791)-13myc*	Davidson and Brown 2008
pRS315-*elg1(NLS-216-731)-13myc*	*amp^r CEN6 ARSH4 LEU2 P_{ELG1}-elg1(NLS-216-731)-13myc*	This study
pRS315-*elg1(NLS-216-519)-13myc*	*amp^r CEN6 ARSH4 LEU2 P_{ELG1}-elg1(NLS-216-519)-13myc*	Davidson and Brown 2008
pRS315-*elg1(NLS-520-791)-13myc*	*amp^r CEN6 ARSH4 LEU2 P_{ELG1}-elg1(NLS-520-791)-13myc*	This study
pTK23	*amp^r NHP6B TRP1 P_{POL30}-POL30*	This study
pTK24	*amp^r NHP6B LEU2 P_{POL30}-POL30*	This study
pTK26	*amp^r NHP6B LEU2 P_{POL30}- pol30-K164R*	This study
pTK27	*amp^r NHP6B LEU2 P_{POL30}-pol30-K127R*	This study
pTK28	*amp^r NHP6B LEU2 P_{POL30}-pol30-K127R&K164R*	This study
pTK30	*amp^r NHP6B LEU2 P_{POL30}-pol30-D150E*	This study
pTK31	*amp^r natNT2 P_{SIC1}-sic1(1-105aa)*	This study
pRDK1597	*amp^r natNT2 P_{CLB6}-clb6(1-195aa)*	Hombauer et al. 2011
pRDK1598	*amp^r natNT2 P_{CLB2}-clb2(1-181aa, L26A)*	Hombauer et al. 2011
pRS303-*GALp*	*amp^r LEU2 $P_{GAL1-10}$*	This study
pRS303-*GALp-POL30-ADH1t*	*amp^r LEU2 $P_{GAL1-10}$-POL30-T_{ADH1}*	This study
